# Color Darkening and Pyrazine Accumulation Associated with Apparent Roasting Intensity and Aroma Quality in Commercial Sesame Paste

**DOI:** 10.3390/foods15183225

**Published:** 2026-09-11

**Authors:** Sirou Gu, Ping Zhan, Zhonghong Wu, Peng Wang, Honglei Tian

**Affiliations:** 1 College of Food Engineering and Nutritional Science, Shaanxi Normal University, Xi’an 710119, China; 2 Institute of Agro-Products Processing, Xinjiang Key Laboratory of Processing and Preservation of Agricultural Products, Xinjiang Academy of Agricultural Science, Urumqi 830091, China; 3 Xinjiang Changji Agricultural Science and Technology Park Agricultural Science and Technology Development Co., Ltd., Changji 831100, China

**Keywords:** sesame paste, apparent roasting intensity, aroma-active compounds, pyrazines, aroma addition

## Abstract

Commercial sesame paste exhibits substantial variations in color and aroma, reflecting differences in raw materials and commercial processing conditions, including roasting. This study investigated 15 commercial sesame paste products to characterize associations among color/browning characteristics, sensory attributes, and aroma-active chemical profiles. Across the commercial samples, darker color and higher browning index (BI) values were generally associated with stronger roasted nutty and burnt attributes and weaker grassy notes. Higher pyrazine levels were also observed in samples exhibiting more pronounced apparent roasting-related aroma characteristics and were positively associated with roasted nutty and burnt attributes. GC-O and OAV screening, together with exploratory PLSR further analysis, indicated that Strecker aldehydes, pyrazines, and phenolic compounds were candidate contributors to the observed sensory differences. In aroma addition experiments, under the tested addition conditions, the R mixture containing 2-methylbutanal and selected pyrazines enhanced roasted nutty intensity, whereas the B mixture containing guaiacol and 4-vinyl-2-methoxyphenol enhanced burnt/smoky intensity under the tested addition conditions. These findings demonstrate that color/browning characteristics and aroma-active compound profiles provide complementary information for characterizing apparent roasting-related differences and aroma quality in commercial sesame paste, without representing direct measures of controlled roasting degree.

## 1. Introduction

Sesame paste is a semisolid condiment produced by roasting and grinding sesame seeds (*Sesamum indicum* L.) and is valued for its characteristic nutty and fatty aroma [1,2,3]. It is widely consumed in East Asia, the Mediterranean region, and the Middle East [4]. Variations in raw materials and processing conditions, particularly roasting temperature and duration, markedly affect the color, aroma, and physicochemical properties of sesame paste [5,6,7]. However, because commercial sesame paste products may differ in raw materials and processing conditions, differences in color and aroma between products cannot be attributed solely to actual roasting degree. Therefore, the relationships among color/browning characteristics, sensory attributes, and aroma-related chemical profiles in commercial sesame paste require further clarification.

Roasting is regarded as a critical processing step that affects color development and aroma formation during sesame paste production [8,9]. Color development and aroma formation may proceed through partly different thermal reaction pathways [5,8,9,10,11]. Color darkening is mainly associated with the formation and accumulation of melanoidins and other products generated during nonenzymatic browning reactions, particularly the Maillard reaction [5,9,12], whereas aroma development involves lipid oxidation, Strecker degradation, the Maillard reaction, and related thermal reactions [8,10,11,13]. Volatile compounds in roasted sesame products are closely associated with roasted, nutty, burnt, fatty, and grassy aroma attributes in sesame paste [2,8,11]. Among roasting-related volatile compounds, pyrazines are particularly important for the formation of the roasted and nutty aroma in sesame products. Pyrazines often show high odor activity values (OAVs) or strong aroma activity and are therefore regarded as important aroma-active compounds in roasted sesame products [8,14,15]. Therefore, color/browning characteristics and aroma-related chemical profiles may provide complementary information regarding roasting-associated changes in sesame paste. Because color and aroma arise from partially distinct reaction pathways and are sensitive to raw-material and processing conditions, their responses may not necessarily be synchronous [3,7,10,11].

Color analysis, sensory evaluation, and instrumental analysis are commonly combined to characterize relationships among color variation, sensory attributes, and volatile aroma compounds in roasted foods [16,17,18]. Color parameters such as $L^{*}$, $a^{*}$, $b^{*}$, ∆E, and the browning index (BI) provide information on browning-related changes during roasting or thermal processing [5,16,17,18]. In a sesame oligosaccharide protein Maillard reaction model, increased roasting temperature and duration were accompanied by greater color difference and browning intensity, while comparable roasting-associated decreases in L and changes in a, $b^{*}$, or BI have also been reported in other roasted food matrices [5,16,17,18]. Descriptive sensory analysis has revealed changes in roasted sesame, nutty, fermented, and green aroma attributes during sesame paste storage [2]. E-nose analysis can characterize overall aroma-response differences among samples but cannot identify individual aroma-active compounds. Therefore, volatile compound composition and sensory contribution of volatile compounds still need to be further elucidated at the molecular level. Headspace solid phase microextraction gas chromatography mass spectrometry (HS-SPME-GC-MS), gas chromatography olfactometry (GC-O), and odor activity value (OAV) analysis have been widely applied to characterize volatile composition and screen compounds with potential aroma activity in sesame paste, peanut butter, pistachio oil, and other oil-rich food systems [2,19,20]. In sesame paste during storage, SAFE-GC-O-MS combined with descriptive sensory analysis identified aroma-active compounds related to nutty, roasted, grassy, and fermented attributes [2]. In oil-based condiments, including fried shallot oil and fried huajiao oil, GC-MS or GC-O combined with OAV and sensory analysis characterized aroma-related volatile compounds and their associations with sensory attributes [21,22]. Aroma addition, recombination, or omission experiments have been applied to further validate the actual sensory contribution of candidate compounds with OAV > 1 in Pu-erh tea, duck broth, Douchi, and flaxseed milk [23,24,25,26].

Multivariate statistical analysis can further support the interpretation of aroma differences by linking chemical variables with sensory perception. Partial least squares regression (PLSR) is commonly used to establish relationships between volatile compounds or aroma-active compounds and sensory attributes. In sesame paste during storage, PLSR linked pyrazines, aldehydes, and phenolic compounds with roasted sesame aroma, nutty aroma, and grassy attributes [2]. In ginger-infused stewed beef and fried coriander oil prepared at different frying temperatures, PLSR has also been used to clarify relationships between volatile compounds and sensory attributes and further support aroma quality evaluation [27,28]. Therefore, PLSR may help explore associations between aroma-active compounds and sensory attributes.

Although volatile compounds and aroma-active compounds in sesame oil or sesame paste have been investigated, limited information is available on the integrated relationships among color/browning characteristics, sensory attributes, overall aroma-response profiles, and aroma-active compound profiles in commercial sesame paste exhibiting different apparent roasting characteristics. The present study investigated 15 commercial sesame paste samples exhibiting broad variation in color and aroma characteristics. Color measurement, QDA, and E-nose analysis were used to characterize color variation, sensory attributes, and overall aroma characteristics of the samples. Volatile aroma compounds were identified by HS-SPME-GC-MS, and aroma-active compounds were screened by GC-O and OAV analysis. PLSR was further applied to explore associations between aroma-active compounds and sensory attributes. Aroma addition validation was conducted to evaluate the sensory contribution of candidate roasting-related compounds. The findings may provide a theoretical basis for flavor quality evaluation, key aroma-active compound identification, and characterization of apparent roasting-related differences and aroma quality in commercial sesame paste.

## 2. Materials and Methods

### 2.1. Samples and Chemicals

**Figure 1:**
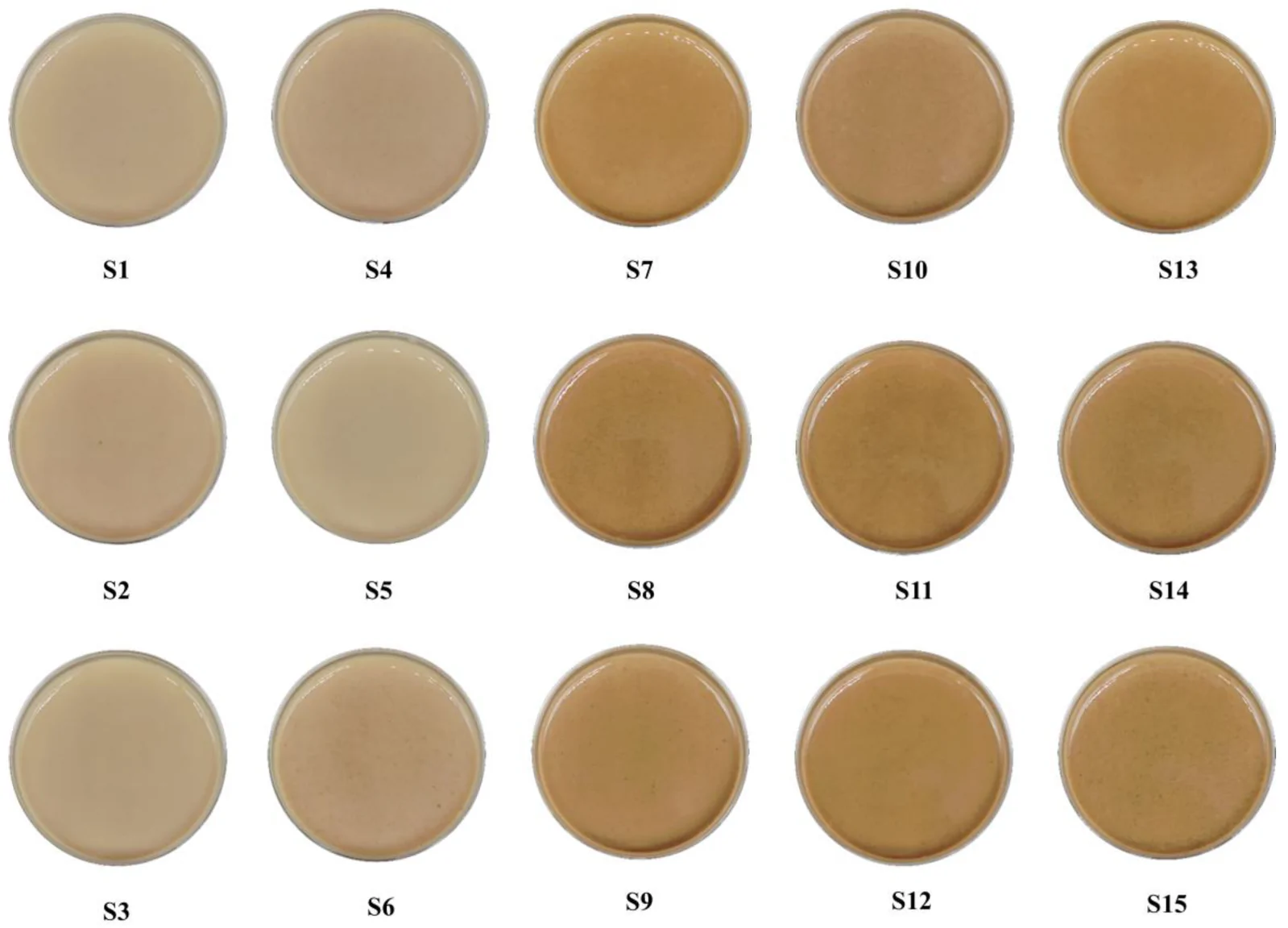
Representative images of the 15 commercial sesame paste samples. The colors shown represent the actual appearance of the samples; quantitative color parameters were determined separately as described in Section 2.3.

The geographic origins of the 15 commercial sesame paste samples are listed in Table S1, and representative photographs are shown in Figure 1, with sample codes arranged sequentially from S1 to S15. Detailed roasting temperature, duration, and manufacturer-defined roasting classifications were not available for the commercial products. Therefore, the samples were not regarded as experimentally controlled roasting treatments, and the observed differences were interpreted in terms of apparent roasting-related characteristics rather than actual roasting degree. After arrival, the samples were immediately stored at 4 °C in the dark to reduce the loss or oxidation of volatile flavor compounds. All experiments were completed within 25 days after sample receipt, and the remaining shelf life of all samples exceeded 10 months. All samples were opened and analyzed on the same day following the same analytical sequence, including color measurement, sensory evaluation, electronic nose analysis, and HS-SPME-GC-MS analysis. Chromatographic grade standards were used for compound identification, including 2-methylbutanal, α-pinene, hexanal, 2-pentylfuran, 2-methylpyrazine, 4-isopropyltoluene, (*E*)-2-octenal, 1-hexanol, 2-ethyl-6-methylpyrazine, 2-ethyl-5-methylpyrazine, 2,3,5-trimethylpyrazine, nonanal, 2-ethyl-3,5-dimethylpyrazine, 1-octen-3-ol, 3-ethyl-2,5-dimethylpyrazine, 1-heptanol, benzaldehyde, 1-octanol, 2-pentylpyridine, phenylacetaldehyde, guaiacol, γ-undecalactone, 4-ethyl-2-methoxyphenol, and 4-vinyl-2-methoxyphenol. All standards were purchased from Sigma-Aldrich (Shanghai, China), Shanghai Aladdin Biochemical Technology Co., Ltd. (Shanghai, China), and Shanghai Yuanye Bio Technology Co., Ltd. (Shanghai, China). 1,2-dichlorobenzene was used as the internal standard (IS) for semi-quantitative estimation of volatile compounds. A homologous series of n-alkanes (C7–C40) was used to calculate the retention indices (RIs) of compounds. Both were purchased from Shanghai ANPEL Laboratory Technologies Inc. (Shanghai, China). The purity of all chemicals was not less than 97%.

### 2.2. Preparation of Sesame Paste Samples

Sesame paste samples stored at 4 °C were thoroughly stirred and then allowed to equilibrate to room temperature. Before measurement, the samples were stirred again to ensure homogeneity.

### 2.3. Determination of Color Difference

Color parameters of the 15 sesame paste samples were determined using an NS810 spectrophotometer (3nh, Shenzhen ThreeNH Technology Co., Ltd., Shenzhen, China) with a d/8° illumination/observation geometry, CIE standard illuminant D65, a 10° standard observer, and an 8 mm measurement aperture [17]. Before measurement, the instrument was calibrated with standard black and white calibration plates. Each sample was placed evenly in the sample container to obtain a flat surface, and $L^{*}$, $a^{*}$, and $b^{*}$ values were recorded at room temperature. $L^{*}$ indicates lightness, $a^{*}$ indicates the red–green axis, and $b^{*}$ indicates the yellow–blue axis. Measurements were performed in triplicate, and mean values were used for further analysis. The sample with the highest $L^{*}$ value was selected as the reference sample for the calculation of ∆E. BI was calculated using the following formula:

(1) $$∆E=\sqrt{{\left({∆L}^{*}\right)}^{2}+{\left({∆a}^{*}\right)}^{2}+{\left({∆b}^{*}\right)}^{2}}$$

(2) $$BI=\frac{\left[100\times \left(\frac{a_{i}^{*}+1.75L_{i}^{*}}{{5.645L}_{i}^{*}+a_{i}^{*}-{3.012b}_{i}^{*}}0.31\right)\right]}{0.17}$$

where ${∆a}^{*}$, ${∆b}^{*}$, and ${∆L}^{*}$ represent the differences in the corresponding color parameters between each sample and the sample with the highest $L^{*}$ value, respectively. $a_{i}^{*}$, $b_{i}^{*}$, and $L_{i}^{*}$ represent the color parameters of each sample.

### 2.4. Quantitative Descriptive Analysis

The aroma profiles of sesame paste samples were assessed by QDA. Descriptive aroma attributes were selected based on the aroma characteristics of sesame paste and the relevant literature [2,7,8]. Five descriptive aroma attributes were used to evaluate the sesame paste samples, including “roasted nutty”, “burnt”, “typical sesame”, “grassy”, and “fatty”. Attribute definitions, reference samples, and scoring criteria are presented in Table S2. The sensory panel comprised 13 trained panelists with previous sensory evaluation experience, including three males and 10 females. Panelists were further trained in aroma attribute recognition and intensity scaling before sensory evaluation. Before evaluation, all panelists were required to avoid spicy, strongly flavored, or irritating foods within 6 h before the test [29]. Each sample, approximately 10.0 g, was placed in a 40 mL capped amber glass vial and coded with a 3-digit random number. Before sniffing, all samples were placed in a water bath initially preheated to 60 °C for 10 min, without further heating during this period, to promote the release of volatile compounds. Similar temperature-controlled conditioning before sensory aroma evaluation has been reported for sesame paste and lipid-rich seasoning oils [3,30,31]. The coded samples were randomly shuffled before evaluation and then presented sequentially according to the resulting order in a temperature-controlled sensory laboratory at 25 °C, with each panelist seated in an individual booth under standardized lighting. For each commercial sesame paste sample, three parallel samples were prepared from three packages of the same product. Each panelist evaluated the three parallel samples separately, and the scores obtained from the three parallel samples were averaged for each panelist before subsequent statistical analysis. Six samples were evaluated in each session, with a 90 s interval between two consecutive samples to reduce carryover effects [29]. During each interval, panelists were instructed to breathe fresh air. No palate cleansers or odor-neutralization procedures were used. A 0–9 scale was used, with higher scores indicating stronger sensory attribute intensity. The final sensory score was expressed as the mean score of all panelists.

### 2.5. E-Nose Analysis

The overall aroma profiles of sesame paste samples were analyzed using an E-nose system equipped with 18 sensors (Super Nose-18, ISENSO, New York, NY, USA) [14,29]. Before measurement, the E-nose system was preheated. Baseline stabilization and sensor cleaning were then performed using clean air. Sample measurement was started after the response signals of all sensors had stabilized. For each measurement, 10.0 g of sample was placed in a 40 mL headspace vial and sealed. The sample was equilibrated in a water bath at 60 °C for 20 min to promote the release and equilibration of volatile compounds. During measurement, the equilibrated headspace gas was introduced into the E-nose detection system using the carrier gas. The carrier gas flow rate was set at 1 L/min, and the injection time was 120 s. After each measurement, the sensors were cleaned for 120 s to reduce carryover effects between samples. All samples were measured in a random order, and each sample was analyzed in quadruplicate. For PCA, the four replicate responses of each sensor were averaged, and the resulting 18 mean sensor responses for each sample were used as input variables.

### 2.6. HS-SPME-GC-MS Analysis

Volatile compounds in commercial sesame paste samples were extracted by HS-SPME. 5.0 g of sample was accurately weighed into a 20 mL headspace vial, and 1.0 μL of 1,2-dichlorobenzene working solution, prepared by diluting the stock solution (1.306 g/mL in methanol) 10,000-fold, was added as the IS for semi-quantitative estimation of volatile compounds. The vial was immediately sealed with Parafilm M film (Amcor Flexibles North America, Neenah, WI, USA). Volatile compounds were enriched using a CAR/DVB/PDMS fiber (50/30 μm, Supelco, Bellefonte, PA, USA). Before use, the fiber was conditioned in the GC inlet for 20 min to remove impurities and stabilize extraction performance. Before sample analysis, an empty-vial procedural blank was subjected to the same HS-SPME-GC-MS procedure, and a fiber blank was analyzed after fiber conditioning to check for background contamination and potential fiber-derived interferences. No additional QC samples were interspersed within the analytical sequence. After equilibration in a water bath at 60 °C for 15 min, the conditioned fiber was inserted into the headspace vial and exposed to the headspace above the sample. Extraction was performed at 60 °C for 30 min. After extraction, the fiber was immediately inserted into the GC inlet and desorbed at 250 °C for 7 min, allowing the adsorbed volatile compounds to be fully desorbed and introduced into the GC-MS system for analysis.

Volatile compounds were analyzed using a GC-MS system (Agilent 7890B-5977B, Agilent Technologies, Santa Clara, CA, USA) equipped with a DB-WAX capillary column (30 m × 0.25 mm × 0.25 μm, Agilent Technologies, Santa Clara, CA, USA). High-purity helium was used as the carrier gas at a flow rate of 1.0 mL/min. The GC temperature program was adapted from the method described by Yang et al., with slight modifications [2]. The initial oven temperature was set at 35 °C and held for 2 min. The temperature was then increased to 185 °C at 5 °C/min and held for 8 min. The MS conditions were as follows: transfer line temperature, 250 °C; ion source temperature, 230 °C; electron impact ionization energy, 70 eV; and mass scan range, *m*/*z* 30 to 450. Each sample was analyzed in triplicate for qualitative identification, semi-quantitative, and statistical analyses of volatile compounds.

### 2.7. GC-O Analysis

Aroma-active compounds in sesame paste samples were analyzed by GC-O [22]. The analysis was performed using a GC system equipped with an olfactory detection port (ODP4, GERSTEL, Mülheim (Ruhr), Germany). The chromatographic effluent was split at a volume ratio of 1:1 into the MS detector and the olfactory detection port. The GC-MS conditions were the same as those described in Section 2.6. During sniffing, the panelists continuously evaluated the chromatographic effluent at the olfactory detection port. The retention time, aroma description, and aroma intensity of each perceivable aroma were recorded. To reduce the influence of nasal mucosa dryness on sniffing performance, humidified air was continuously supplied to the olfactory detection port at a flow rate of 40 mL/min. GC-O analysis was performed by three panelists with previous sniffing experience, and each sample was analyzed twice. A compound was considered aroma-active when the corresponding odor was detected by at least two of the three panelists.

### 2.8. Identification and Quantitative Analysis of Aroma-Active Compounds

Volatile compounds were identified based on multiple criteria, including the mass spectral database (NIST 14.0), retention indices (RIs), and standards. The overall volatile profile was characterized semi-quantitatively using 1,2-dichlorobenzene as an internal reference. The RIs were determined using a homologous series of n-alkane standard solutions (C_7_–C_40_) and calculated according to the method described by Pang et al. [32]. Aroma-active compounds for which authentic standards were available were quantified using five-point external standard calibration curves. Calibration curves were constructed by plotting the concentration ratio of each target compound to the IS as the x-axis and the corresponding peak area ratio as the y-axis [33]. These calibration curves were used for the quantitative calculation of each aroma-active compound. The calculated concentrations of these aroma-active compounds were subsequently used for OAV calculation.

### 2.9. Calculation of OAV

OAV was used to evaluate the potential contribution of each volatile compound to the overall aroma and was calculated according to reported methods [21,22]:

(3) $$OAV=\frac{C_{i}}{{OT}_{i}}$$

where C_i_ represents the quantified concentration of compound i, and OT_i_ represents the corresponding odor threshold of compound i. Considering the lipid-rich characteristics of sesame paste, odor thresholds determined in oil-based media were preferentially selected for OAV calculation. When oil-based odor thresholds were unavailable, reported thresholds determined in aqueous media were used as alternatives. Further details on the threshold media and OAV results are provided in Section 3.5. The OT_i_ values of the compounds were obtained from published literature [34]. An OAV > 1 was used as a screening criterion to identify compounds with potential aroma contributions. Therefore, OAVs were used as screening indices for potential aroma contribution rather than as absolute measures of sensory contribution, particularly for compounds whose odor thresholds were obtained in aqueous media.

### 2.10. Aroma Addition Experiment

Aroma addition, recombination, and omission experiments have been widely used to evaluate the sensory effects of candidate aroma-active compounds in food matrices [24,25,26]. To evaluate the effects of candidate aroma-active compounds on the sensory attributes of sesame paste, a sample exhibiting relatively weak apparent roasting-related aroma characteristics was selected as the base matrix for the aroma addition experiment. Based on the GC-O sniffing results, OAV analysis, and exploratory PLSR analysis, representative compounds with potential aroma contributions were selected and prepared as different types of aroma compound mixtures. The selected compounds were assigned to the R mixture, B mixture, or combined R+B mixture according to the sensory attributes targeted in the addition experiment. The identity of the base matrix and the identities and exact supplemental concentrations of the selected compounds are provided in Table S3. A blank control group (CK, matrix sample with carrier), an R mixture group, a B mixture group, and a combined R+B mixture group were established. For each treatment, 10.00 g of the selected base matrix was used. The CK group received 100 μL of deodorized vegetable oil, whereas the other groups received 100 μL of the corresponding aroma mixture prepared in deodorized vegetable oil. After addition, the samples were immediately capped, thoroughly mixed for 2–3 min, and equilibrated at room temperature in the dark for 1 h before sensory evaluation. The treated samples were evaluated by QDA. The panelists scored the intensities of “roasted nutty”, “burnt”, “typical sesame”, “grassy”, and “fatty” attributes. The addition groups were compared with CK to evaluate changes in the corresponding sensory attributes following aroma addition.

### 2.11. Statistical Analysis

Hierarchical cluster analysis based on color parameters and one-way analysis of variance (ANOVA) of color and sensory data followed by Tukey for multiple comparisons were performed using SPSS 27.0 software (IBM Corp., Armonk, NY, USA). Data visualization of sensory attributes, E-nose responses, volatile compounds, GC-O results, aroma addition results, and Pearson correlation analysis among color parameters, sensory attributes, and pyrazine compounds was performed using Origin 2024 (OriginLab Corporation, Northampton, MA, USA). Heatmap visualization and hierarchical clustering of volatile compounds and pyrazine compounds were performed using TBtools-II (South China Agricultural University, Guangzhou, Guangdong, China, China). Before hierarchical clustering, volatile compound concentrations were log_2_(x + 1)-transformed and standardized by row using Z-score scaling. Hierarchical clustering of samples was subsequently performed using Euclidean distance and complete linkage. PLSR analysis between sensory attributes and aroma-active compounds was performed using The Unscrambler X 10.4 software (CAMO ASA, Oslo, Norway). For each commercial sesame paste product, three separate packages were purchased and analyzed to characterize within-product variability, and the results were expressed as mean ± standard deviation. For cross-product correlation analysis and PLSR, product-level mean values were used, resulting in 15 independent observations (*n* = 15). The three packages within each product were treated as package-level replicates and were not used to increase the product-level sample size. The replication design for each analytical method is described in the corresponding sections. The significance level was set at *p* < 0.05.

## 3. Results and Discussion

### 3.1. Color and Browning Characteristics of Commercial Sesame Paste Samples

The color parameters ($L^{*}$, $a^{*}$, and $b^{*}$) and BI values of the 15 commercial sesame paste samples are shown in Table 1. Significant differences were observed in $L^{*}$, $a^{*}$, $b^{*}$, and BI values among different samples (*p* < 0.05). The color analysis results indicated marked color differences among commercial sesame paste samples. Sample S5 had the highest $L^{*}$ value and showed the lightest paste color. Sample S11 had the lowest $L^{*}$ value and showed the darkest paste color. The $a^{*}$ and $b^{*}$ values generally increased as $L^{*}$ decreased. The change indicated a gradual shift toward reddish yellow or brown tones with decreasing sample lightness. The ΔE values were calculated using sample S5 as the reference because sample S5 had the highest $L^{*}$ value. The ΔE results indicated pronounced color differences among commercial sesame paste samples. Most color differences could be visually distinguished [35]. BI generally increased with decreasing L*, indicating greater browning in darker samples. Sample darkening may be related to enhanced browning reactions during roasting [9]. Similar roasting-induced darkening has also been reported in peanut kernels, Chinese hybrid hazelnuts, and shelled macadamia kernels [16,17,18]. During roasting, the Maillard reaction and caramelization can promote the formation of melanoidins and other brown products, thereby contributing to color darkening [5,9,12].

To characterize variation in the color/browning dimension of the commercial sesame paste samples, hierarchical cluster analysis was performed. The analysis was based on $L^{*}$ and BI values of 15 commercial sesame paste samples (Figure S1). The first cluster comprised light color and low browning samples (S1–S6). Samples in the first group had higher $L^{*}$ values and lower BI values. The second cluster comprised samples with medium-to-dark color and generally higher browning indices (S7–S15). Compared with the first cluster, samples in the second cluster generally showed lower $L^{*}$ values and higher $a^{*}$, $b^{*}$, and BI values. The clustering result indicated that L* and BI could effectively describe differences in the color/browning dimension of the commercial samples but should not be regarded as definitive indicators of roasting intensity [5,9,16]. Color differences among samples may be associated with variations in browning reactions and color formation during roasting [5,9,12]. However, because commercial products were analyzed, the observed color differences could not be attributed solely to roasting intensity.

Hierarchical clustering based on $L^{*}$ and BI mainly separated the samples into two groups. The clustering result did not fully distinguish samples exhibiting intermediate and stronger apparent roasting-related characteristics, indicating that fine characterization of apparent roasting-related differences based only on color indicators remained limited. Apparent roasting-related differences may also affect the aroma characteristics of sesame paste samples [2,8]. Therefore, color parameters alone were insufficient to represent the overall roasting-associated characteristics of commercial sesame paste, and aroma-related sensory and chemical responses were subsequently evaluated as a complementary dimension.

### 3.2. Sensory Analysis of Different Types of Sesame Paste Samples

**Figure 2:**
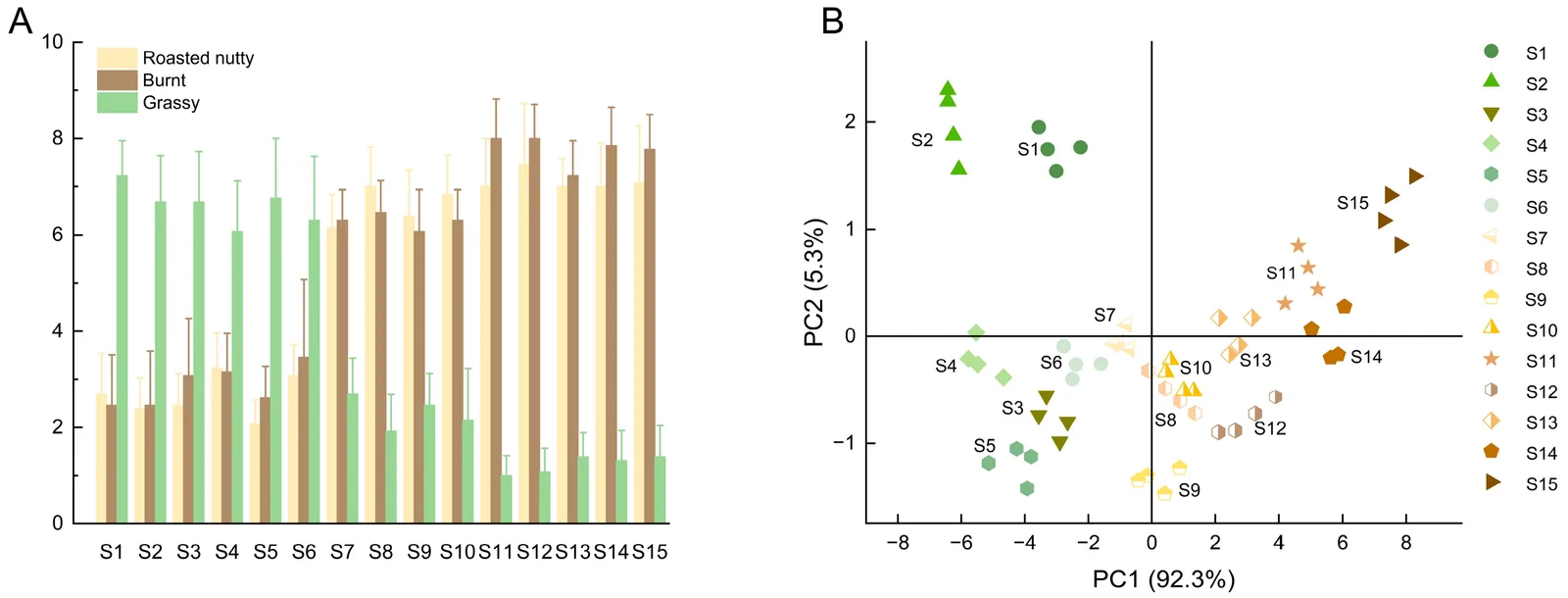
(**A**) Scores of three sensory attributes of sesame paste samples. (**B**) PCA of commercial sesame paste samples based on electronic nose responses.

QDA was used to evaluate the aroma characteristics of the 15 commercial sesame paste samples. QDA results are shown in Table 2. Changes in roasted nutty, burnt, and grassy attributes are shown in Figure 2A. Clear differences in aroma attributes were observed among different samples. Significant differences were observed among the 15 commercial samples for all five sensory attributes (*p* < 0.05).

Combined with the color clustering result described above, samples S1–S5 generally showed higher grassy intensity and weaker roasting-related aroma characteristics. The sensory scores indicated that samples S1–S5 were mainly characterized by grassy aroma derived from raw materials. Sample S6 belonged to the same light color and low browning group as samples S1–S5 in the color clustering result. However, S6 exhibited relatively stronger roasted nutty, burnt, and fatty characteristics than most samples in S1–S5, suggesting that its color/browning and aroma responses were not completely synchronous. In contrast, samples S7–S15 showed stronger roasting-related aroma characteristics and lower grassy intensity, with S11–S15 generally exhibiting more pronounced burnt and fatty attributes than S7–S10. The sensory changes indicated that samples S11–S15 had more pronounced apparent roasting-related aroma characteristics. Samples S7–S10 and S11–S15 still showed some overlap in roasted nutty and fatty attributes. Therefore, QDA results provided complementary information on sensory variation among the commercial samples.

Overall, the grassy attribute gradually decreased as sample color deepened. Roasted nutty, burnt, and fatty attributes gradually increased. The increases in roasted nutty and burnt attributes may be associated with the Maillard reaction, Strecker degradation, and lipid thermal oxidation during roasting [5,8,10,13]. These reactions can promote the formation of pyrazines, furans, and other volatile compounds associated with roasted and burnt aroma characteristics [8,10,13,36,37]. The decrease in grassy attribute may be related to the transition from raw material-derived aroma characteristics to roasting-dominated aroma characteristics during roasting [8,38]. Enhanced roasted aroma may also weaken the relative perception of grassy aroma to some extent. QDA further characterized sensory differences among the commercial samples and complemented the color/browning analysis in describing apparent roasting-related patterns.

### 3.3. E-Nose Response Profiles and PCA

To further evaluate differences in the overall aroma profiles of sesame paste samples, E-nose combined with principal component analysis (PCA) was used to analyze the 15 samples. The PCA score plot is shown in Figure 2B. PC1 and PC2 explained 92.3% and 5.3% of the total variance, respectively. The cumulative contribution rate reached 97.6%. Therefore, the first two principal components captured most of the variation in the E-nose response data.

The PCA score plot showed clear distribution differences among the 15 samples. Samples S1–S6 were mainly distributed in the negative direction of PC1. Samples S7–S15 were mostly distributed in the positive direction of PC1. Sample S6 was located near the transitional region between samples S1–S5 and samples S7–S15. The location of sample S6 was consistent with the partial divergence between its color and aroma-related characteristics observed in sensory evaluation. Overall, the PCA results revealed variation in the overall aroma-response profiles of the commercial sesame paste samples rather than providing an independent basis for sample classification. However, E-nose analysis reflects overall aroma responses but cannot identify individual aroma-active compounds. Therefore, HS-SPME-GC-MS, GC-O, and OAV analyses were further conducted to characterize volatile and aroma-active compounds [8,39]. Together with the color and sensory results, the E-nose responses supported the presence of systematic apparent roasting-related differences among the commercial samples.

### 3.4. Volatile Compound Composition of Different Types of Sesame Paste Samples and Relationship with Roasting Aroma Characteristics

**Figure 3:**
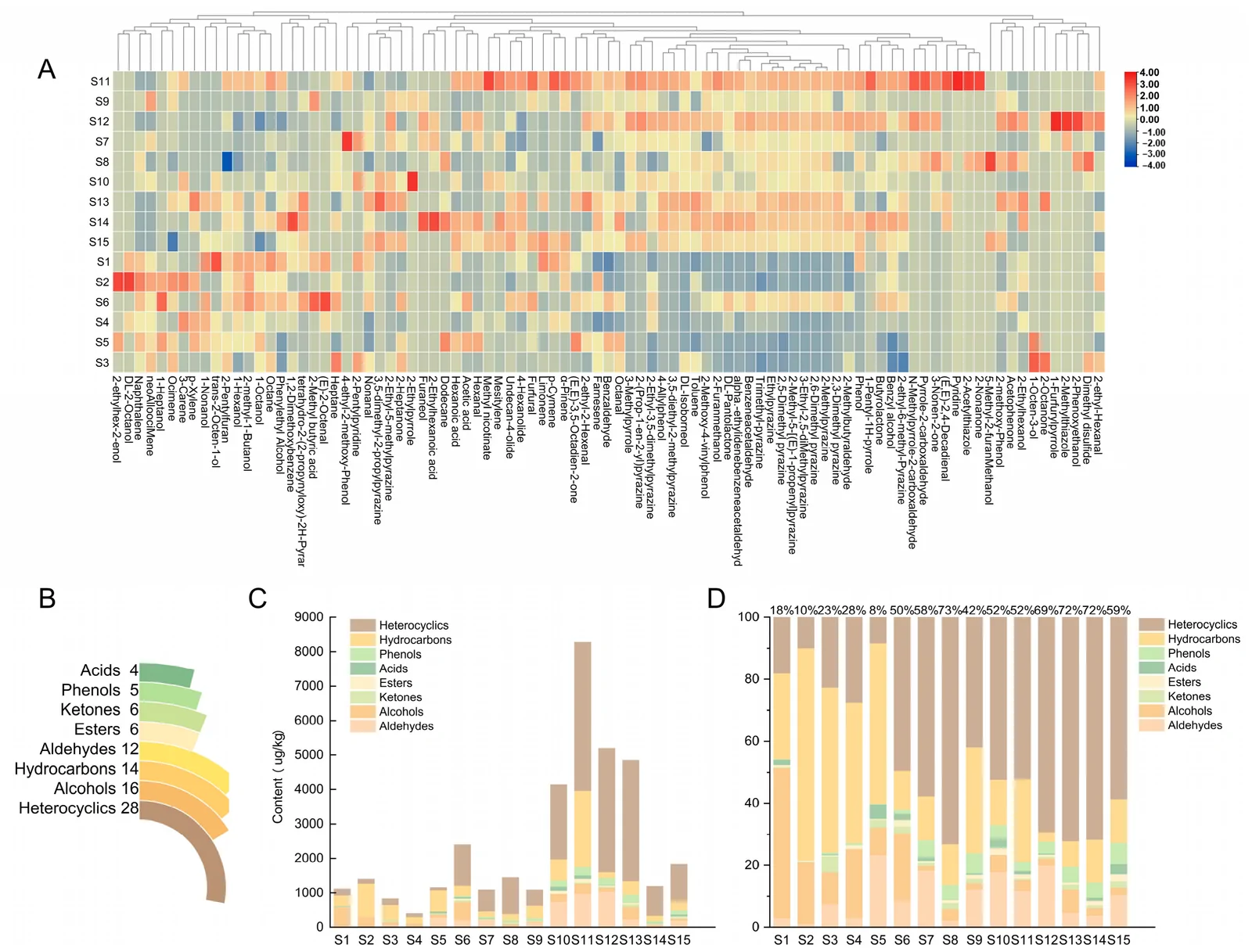
(**A**) Hierarchical clustering analysis based on volatile compound concentrations. (**B**) Composition of volatile compounds in sesame paste samples. (**C**) Content distribution of different volatile compound classes among samples. (**D**) Percentage distribution of volatile compound classes in different sesame paste samples.

HS-SPME-GC-MS was used to analyze volatile compounds in the 15 commercial sesame paste samples. The results are shown in Figure 3. A total of 91 volatile compounds were detected, including 12 aldehydes, 16 alcohols, six ketones, six esters, four acids, five phenols, 14 hydrocarbons, and 28 heterocyclic compounds (Figure 3B). Detailed identification information and relative contents of the detected volatile compounds are provided in Table S4. Among all compound classes, heterocyclic compounds showed the highest number and accounted for 30.77% of all detected compounds. Heterocyclic compounds mainly included pyrazines, furans, pyrroles, pyridines, and thiazoles.

The clustering heat map based on volatile compound concentrations showed clear differences in volatile compound composition among commercial sesame paste samples (Figure 3A). Overall, samples S1–S6 and samples S7–S15 showed a general separation trend in volatile compound distribution, although variation among individual samples was also observed. Samples S11–S14 generally showed higher total volatile compound concentrations and larger proportions of heterocyclic compounds, whereas S1–S5 showed relatively lower levels (Figure 3C,D). Notably, the total volatile compound concentration of sample S6 was 2404.90 μg/kg, higher than the concentrations of most samples in S1–S5. The proportion of heterocyclic compounds in sample S6 reached 50%. Despite its relatively light color and low browning characteristics, S6 exhibited stronger roasting-related characteristics in its volatile profile, indicating that color development and volatile composition were not completely synchronous among the commercial samples. Unlike the more systematic trends commonly observed in controlled roasting studies, the commercial samples showed considerable variation among commercial products, which may reflect differences in raw-material properties, initial moisture content, roasting conditions, subsequent processing, and pre-purchase storage history [8,40].

The main aldehydes detected in the present study included 2-methylbutanal, hexanal, nonanal, and (*E*, *E*)-2,4-decadienal. Relatively high concentrations of 2-methylbutanal were observed in some samples exhibiting stronger apparent roasting-related characteristics, whereas substantially lower levels were generally observed in samples with weaker roasting-related characteristics. 2-Methylbutanal is a typical Strecker aldehyde, and the high concentrations of 2-methylbutanal in S11, S12, and S14 may be related to enhanced Strecker degradation or Maillard reaction-related pathways [13,41]. The variation in hexanal concentration among different samples was not fully consistent with the overall apparent roasting-related pattern. Hexanal is usually associated with lipid oxidation and thermal degradation of unsaturated fatty acids, especially polyunsaturated fatty acids [42,43,44]. The result suggested that hexanal concentration may be affected by thermal processing, lipid oxidation status of raw materials, storage conditions, and processing differences. Nonanal and (*E*, *E*)-2,4-decadienal are mainly related to unsaturated fatty acid oxidation and thermal oxidative degradation [18,42,43]. The concentrations of nonanal in S13, S15, and S6 were 73.57, 42.91, and 30.88 μg/kg, respectively. (*E*, *E*)-2,4-Decadienal was mainly detected in S11, S8, S13, and S14. The changes in aldehydes indicated that lipid oxidation and thermal processing-related reactions may contribute to the formation of the overall aroma profile of sesame paste [10,38]. Alcohols mainly included 1-hexanol, 1-octanol, 1-octen-3-ol, benzyl alcohol, and phenethyl alcohol. Different alcohols can present grassy, fresh, mushroom, floral, or sweet aroma characteristics. High concentrations of 1-hexanol were observed in S1 and S6, with values of 371.30 and 230.40 μg/kg, respectively. 1-Octen-3-ol was mainly detected in S3 and S5, with concentrations of 66.00 and 27.53 μg/kg, respectively. The result suggested that relatively high levels of alcohols related to grassy aroma were retained in some light-colored or transitional samples. In contrast, the relative proportion of alcohols decreased overall in S7–S15. The result indicated that some fresh or grassy volatiles related to raw materials were generally less prominent in samples exhibiting stronger apparent roasting-related characteristics, accompanied by a shift toward roasting-dominated aroma characteristics [15,38]. Several phenolic compounds were also detected in the samples, including guaiacol, phenol, 4-ethyl-2-methoxyphenol, and 4-vinyl-2-methoxyphenol. Phenolic compounds are usually associated with smoky, spicy, burnt, and roasted aroma characteristics [45]. 4-Vinyl-2-methoxyphenol was markedly enriched in several samples exhibiting stronger apparent roasting-related characteristics compared with S1–S5. Based on its smoky, burnt, and roasted aroma characteristics, 4-vinyl-2-methoxyphenol may be an important candidate contributor to the enhanced burnt and smoky characteristics of these commercial samples [15]. Compared with the more systematic changes reported under controlled roasting conditions, greater compound-specific variability was observed among the commercial samples, possibly reflecting differences in raw-material properties, processing conditions, and storage history.

**Figure 4:**
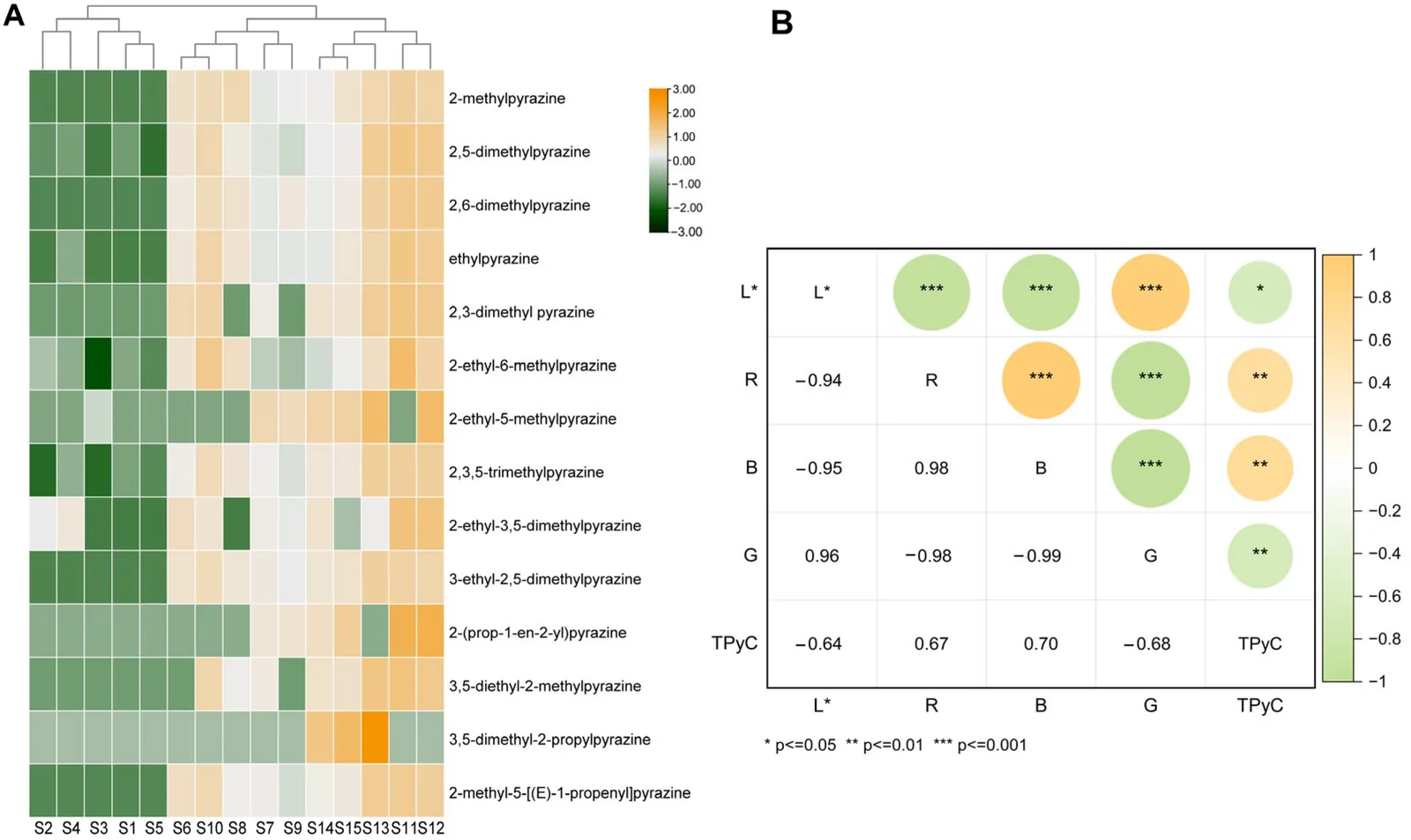
Distribution of pyrazine compounds and their correlations with color and sensory attributes in sesame paste samples. (**A**) Clustered heatmap based on pyrazine compound concentrations; (**B**) Correlation analysis among $L^{*}$ value, sensory attributes, and total pyrazine content (TPyC).

A total of 14 pyrazines were detected in the present study. The main pyrazines included 2-methylpyrazine, 2,5-dimethylpyrazine, 2,3,5-trimethylpyrazine, 2-ethyl-6-methylpyrazine, and 3-ethyl-2,5-dimethylpyrazine. Given that pyrazines are mainly generated through the Maillard reaction and related thermal reaction pathways [38,46] and have low odor thresholds, changes in pyrazine content and composition may provide chemical information related to roasted aroma development in sesame paste [5,47]. As shown in Figure 4A, clear differences in pyrazine distribution were observed among different samples. Samples S1–S5 showed low pyrazine levels overall. The total pyrazine content of S1–S5 ranged from 13.25 to 75.86 μg/kg, with a mean value of only 39.63 μg/kg. In contrast, the total pyrazine content increased markedly in S7–S10, with a mean value of 724.39 μg/kg. The total pyrazine content further increased in S11–S15, with a mean value of 2797.65 μg/kg. The mean value in S11–S15 was approximately 70.6 times higher than the mean value in S1–S5. The total pyrazine contents in S11, S12, and S13 reached 4047.40, 3510.38, and 3390.46 μg/kg, respectively. The results indicated marked differences in pyrazine levels among the commercial samples, with higher levels generally observed in samples exhibiting stronger roasting-related characteristics. Pyrazine accumulation may contribute to the enhancement of roasted aroma characteristics [7,38,46]. Notably, S6 contained substantially higher total pyrazine levels than S1–S5 despite its relatively light color and low browning characteristics. S6 showed relatively light color characteristics but stronger roasted nutty and burnt attributes and substantially higher pyrazine levels than most other light-colored samples. The discrepancy between its color and aroma-related characteristics indicates that the two response dimensions are not necessarily synchronous across commercial products. This observation further suggests that color alone cannot serve as a deterministic measure of roasting intensity in commercial sesame paste. For individual compounds, 2,5-dimethylpyrazine, 3-ethyl-2,5-dimethylpyrazine, and 2,3,5-trimethylpyrazine were representative pyrazines with high concentrations. These representative pyrazines were generally enriched in samples exhibiting stronger apparent roasting-related characteristics. The above pyrazines are usually associated with nutty, roasted, and burnt aroma characteristics [15,38,46]. Similar increases in pyrazine formation have been reported under controlled roasting conditions [5,7,40]. Representative pyrazines may also contribute to the enhanced roasted aroma characteristics of medium- to dark-colored and high-browning samples [15,38,46].

To further clarify the relationships among color characteristics, sensory attributes, and pyrazine accumulation, correlation analysis was performed among $L^{*}$ value, sensory attributes, and total pyrazine content (TPyC) (Figure 4B). Highly significant negative correlations were observed between $L^{*}$ and roasted nutty (R) and burnt (B) (r = −0.94 and −0.95, respectively; *p* < 0.001). A highly significant positive correlation was observed between $L^{*}$ value and grassy attribute (G) (r = 0.96, *p* < 0.001). The grassy attribute decreased. A significant negative correlation was observed between TPyC and $L^{*}$ (r = −0.64, *p* < 0.05). Significant positive correlations were observed between TPyC and roasted nutty and burnt attributes (r = 0.67 and 0.70, respectively; *p* < 0.01). Whereas a significant negative correlation was observed with grassy intensity (r = −0.68, *p* < 0.01). The correlation results indicated that the increase in total pyrazine content was closely associated with the enhancement of roasting-related aroma attributes. In addition, a highly significant positive correlation was observed between roasted nutty and burnt attributes (r = 0.98, *p* < 0.001). Highly significant negative correlations were observed between roasted nutty and grassy attributes and between burnt and grassy attributes (r = −0.98 and −0.99, respectively; *p* < 0.001). The correlation analysis showed that darker commercial samples generally exhibited stronger roasted nutty and burnt attributes and weaker grassy characteristics. Higher pyrazine levels were also associated with stronger roasted nutty and burnt attributes. These relationships represent associations among the measured variables and do not establish direct causal effects of roasting intensity.

Overall, higher pyrazine levels were generally associated with darker color and stronger roasted nutty and burnt attributes across the commercial samples. The results indicated that pyrazines represent a major chemical change accompanying stronger apparent roasting-related characteristics [5,7]. Combined with the present correlation results, pyrazines may also serve as important chemical indicators for evaluating aroma differences among sesame pastes with different apparent roasting-related characteristics.

For descriptive comparisons in the subsequent analyses, the commercial samples were assigned to three data-derived groups according to the overall apparent roasting-related patterns observed across color/browning, sensory, E-nose, and volatile profiles. Samples S1–S5, S6–S10, and S11–S15 were designated as low (L), intermediate (M), and high (H) apparent-roasting groups, respectively. These groups were used only for descriptive comparison and did not represent manufacturer-defined, experimentally controlled, or independently validated roasting treatments.

### 3.5. Analysis of Aroma-Active Compounds in Commercial Sesame Paste Based on GC-O Combined with OAV

**Figure 5:**
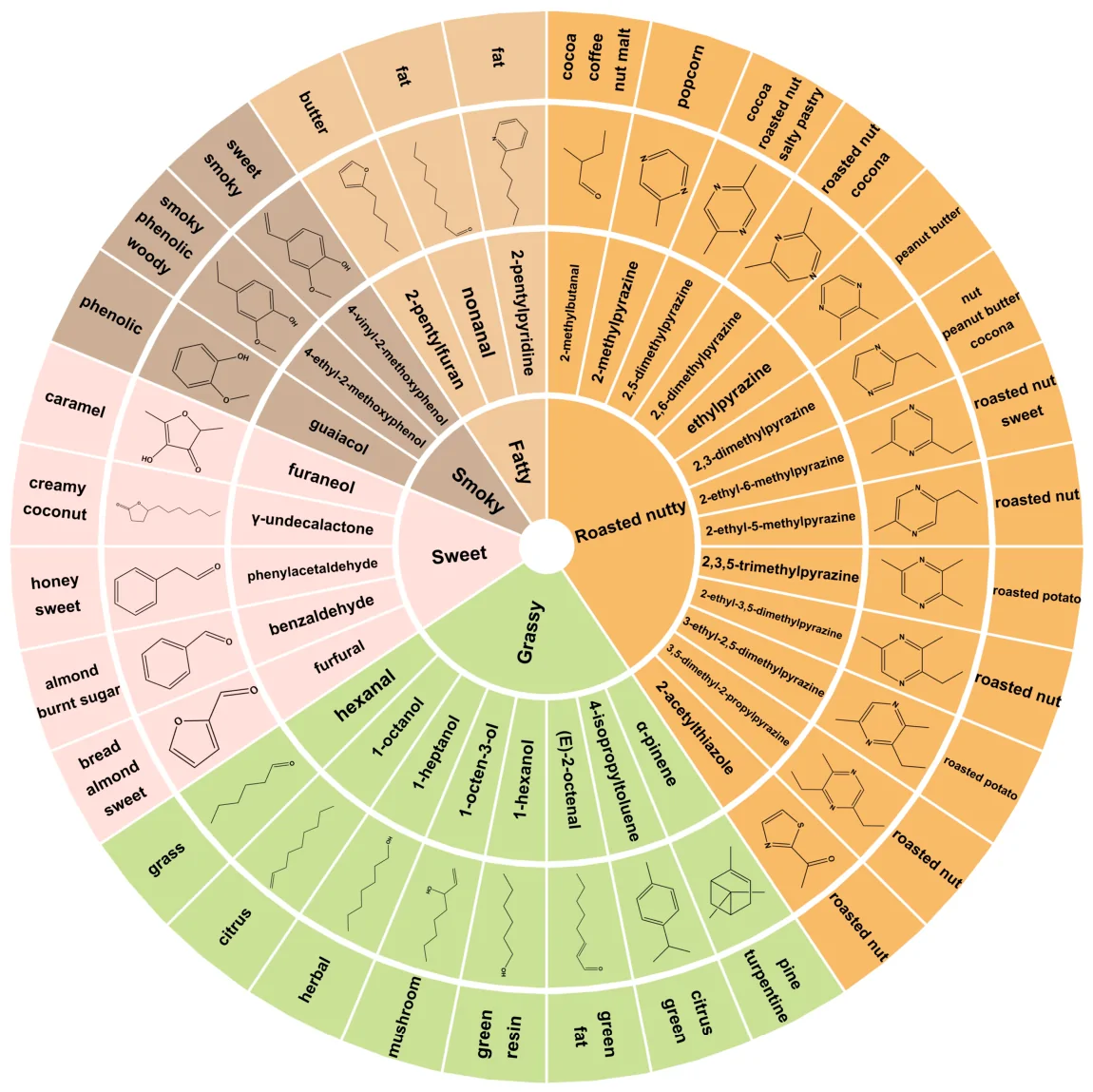
Aroma wheel of aroma-active compounds in sesame paste samples screened by GC-O. From the center outward, the layers represent aroma categories, compound names, chemical structures, and aroma descriptors of the 32 aroma-active compounds. Different colors indicate different aroma categories.

Although many volatile compounds were identified by HS-SPME-GC-MS, volatile abundance alone cannot fully reflect aroma activity [25,48]. Therefore, GC-O was used to screen aroma-active compounds with detectable sniffing responses, and OAV analysis was further applied to estimate their potential aroma contributions [49,50]. A total of 32 aroma-active compounds were screened by GC-O, including seven aldehydes, four alcohols, one ketone, one ester, three phenols, two hydrocarbons, and 14 heterocyclic compounds. Based on sniffing characteristics and sensory descriptors, the 32 aroma-active compounds were classified into major aroma categories, including roasted nutty, grassy, fatty, sweet, and smoky aromas (Figure 5). The aroma wheel showed that the overall aroma of sesame paste was not formed by a single aroma category. The overall aroma was formed by the combined contribution of multiple aroma-active compounds [51,52].

A relatively high proportion of compounds was related to the roasted nutty aroma. The main compounds included pyrazines such as 2-methylpyrazine, 2,5-dimethylpyrazine, 2,3,5-trimethylpyrazine, 2-ethyl-5-methylpyrazine, 2-ethyl-6-methylpyrazine, and 3-ethyl-2,5-dimethylpyrazine. The above compounds mainly presented nutty, roasted, cocoa, popcorn, and peanut butter-like aroma characteristics [15,38,46]. The result suggested that pyrazines may be important candidate contributors to roasted nutty aroma formation in sesame paste [15,38]. Compounds related to grassy and fatty aromas mainly included 1-octen-3-ol, 1-hexanol, (*E*)-2-octenal, hexanal, nonanal, 2-pentylfuran, and 2-pentylpyridine. The above compounds may be related to grassy attributes and lipid oxidation-related aroma in samples exhibiting relatively weak apparent roasting characteristics [38,43]. Compounds related to sweet aroma mainly included furfural, benzaldehyde, phenylacetaldehyde, furaneol, and lactones. Sweet aroma compounds may contribute to caramel-like, sweet, floral, or honey-like aroma characteristics [15,25]. Compounds related to smoky aroma mainly included phenolic compounds such as guaiacol, 4-ethyl-2-methoxyphenol, and 4-vinyl-2-methoxyphenol. Phenolic compounds may be related to stronger burnt and smoky aroma characteristics in samples exhibiting more pronounced apparent roasting characteristics [15].

GC-O can screen aroma-active compounds with detectable sniffing responses, whereas OAV analysis can further estimate the potential contribution of individual compounds to the overall aroma profile [25,49,50]. Therefore, OAV analysis was performed for the 24 GC-O-screened aroma-active compounds for which authentic standards were available. The results showed that 24 compounds had OAV > 1, suggesting potential contributions to the overall aroma of sesame paste (Table 3) [49,50]. Considering the complex sources of commercial sesame paste samples, a compound was included as a candidate aroma-active compound in a given descriptive apparent-roasting group when OAV > 1 was observed in at least one sample within that group. Group means of OAV and the occurrence frequency of OAV > 1 were further used to evaluate the representativeness of candidate compounds. According to the criterion, 20 compounds were retained as candidate aroma-active compounds in at least one descriptive apparent-roasting group.

OAV results showed clear differences in potential aroma-active compound profiles among the three descriptive apparent-roasting groups. In the L group, eight compounds had OAV > 1 in at least one sample. 1-octen-3-ol showed the highest mean OAV in the L group (18.71), but the occurrence frequency of OAV > 1 was low (40%), suggesting a sample-specific contribution. 2-ethyl-3,5-dimethylpyrazine was the main pyrazine related to roasted aroma in the L group, with a mean OAV of 4.57 and an occurrence frequency of 40%. The result indicated that several roasting-related aroma compounds were detected in the L group, although the overall apparent roasting-related aroma characteristics remained relatively weak. In the M group, 14 compounds had OAV > 1 in at least one sample. Compared with the L group, the number, mean OAV, and occurrence frequency of pyrazines were higher in the M group. 2-methylbutanal and 3-ethyl-2,5-dimethylpyrazine showed high potential contributions, with mean OAVs of 58.06 and 8.32, respectively, and both compounds showed an occurrence frequency of 100%. The result indicated that roasted nutty-related compounds showed greater potential aroma contributions in the M group than in the L group. In the H group, 16 compounds had OAV > 1 in at least one sample, representing the highest number among the three groups. The mean OAVs and occurrence frequencies of several pyrazines were also higher in the H group. Pyrazines are usually associated with roasted and nutty aroma characteristics in sesame-related systems [15,38]. The mean OAV of 2-methylbutanal increased to 142.48. The mean OAVs of guaiacol and 4-vinyl-2-methoxyphenol reached 2.44 and 2.80, respectively, and the occurrence frequencies were 60% and 100%, respectively. Phenolic compounds, especially guaiacol and 4-vinyl-2-methoxyphenol, may be associated with the stronger burnt and smoky aroma characteristics observed in samples from the H group [15].

Overall, clear changes in candidate aroma-active compounds were observed among the descriptive apparent-roasting groups. Compounds related to grassy and fatty aromas were more prominent in samples with relatively weak apparent roasting characteristics, whereas pyrazines and phenolic compounds were more prominent in samples with stronger apparent roasting-related aroma characteristics. The changing trend was consistent with the sensory evaluation and volatile compound analysis results described above. The results indicated that pyrazines may provide an important chemical basis for the enhancement of roasted nutty aroma in sesame paste [15,38]. Phenolic compounds may be related to stronger burnt and smoky aroma characteristics in samples from the H group [15]. OAV mainly reflects potential aroma contribution based on compound concentration and odor threshold; therefore, the actual contribution of candidate aroma-active compounds should be further interpreted by PLSR correlation analysis and validated by aroma addition experiments [26,49].

### 3.6. Correlation Analysis Between Aroma-Active Compounds and Sensory Attributes Based on PLSR

To further clarify the relationships between aroma-active compounds and sensory attributes, a PLSR model was established using log(OAV + 1) values of aroma-active compounds as X variables and QDA sensory scores as Y variables. Factor 1 and Factor 2 explained 36% and 19% of the variation in X variables, respectively. The cumulative explained variation in X variables was 55%. Factor 1 and Factor 2 also explained 82% and 9% of the variation in Y variables, respectively. The cumulative explained variation in Y variables reached 91%. The PLSR model was interpreted primarily as an exploratory description of associations between aroma-active compounds and sensory attributes, given the limited number of commercial samples.

**Figure 6:**
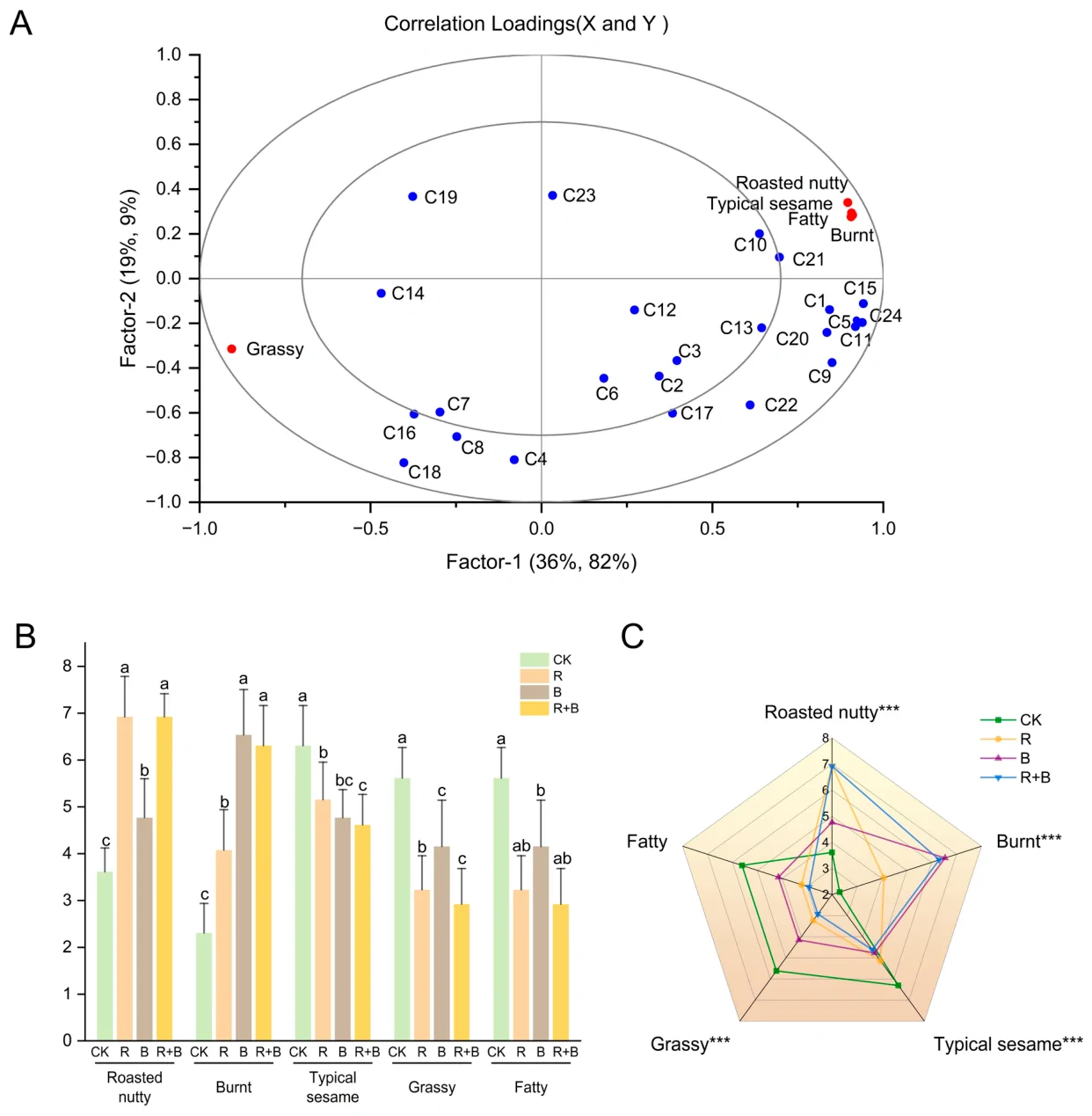
Correlation analysis between aroma-active compounds and sensory attributes and aroma-active compound addition experiment. (**A**) PLSR correlation loading plot of aroma-active compounds and sensory attributes. Blue points indicate aroma-active compounds, red points indicate sensory attributes, and C1–C24 correspond to the compound numbers listed in Table 3 (**B**) Sensory scores of different treatment groups. (**C**) Radar plot of sensory profiles. CK, control group with carrier only; R, roasted nutty aroma-related compound addition group; B, burnt/smoky aroma-related phenolic compound addition group; R+B, combined addition group containing R and B compounds. Different lowercase letters indicate significant differences among treatment groups within the same sensory attribute (*p* < 0.05). In the radar plot, *** indicates highly significant differences among treatment groups for the corresponding sensory attribute (*p* < 0.001).

The PLSR correlation loading plot is shown in Figure 6A. Roasted nutty, burnt, typical sesame, and fatty attributes were distributed on the right side of the plot and were close to each other, indicating positive correlations among these sensory attributes. The grassy attribute was located on the left side of the plot and showed an opposite direction to the roasted nutty and burnt attributes, indicating negative correlations between the grassy attribute and roasting-related attributes. This result was consistent with the sensory evaluation trend described above, with enhanced roasted nutty and burnt attributes and a decreased grassy attribute. Based on the distribution of aroma-active compounds, C1, C5, C9, C10, C11, C15, C20, C21, and C24 were mainly distributed on the right side of the plot and were close to roasted nutty, burnt, typical sesame, and fatty attributes. C1 (2-methylbutanal) and several pyrazines may be related to the enhancement of roasted nutty and typical sesame attributes. Pyrazines have been reported as important contributors to the roasted and nutty aroma in sesame-related products [15,38]. Phenolic compounds, including C21 (guaiacol) and C24 (4-vinyl-2-methoxyphenol), were closer to the direction of the burnt attribute. Phenolic compounds may be related to the enhancement of the burnt and smoky aroma characteristics, especially because 4-vinyl-2-methoxyphenol has been associated with a burnt and smoky aroma in roasted sesame hulls [15]. In contrast, C7, C8, C14, C16, C18, and C19 were mainly distributed on the left side or lower left region of the plot and were close to the direction of the grassy attribute. The distribution suggested possible associations between the listed compounds and grassy or raw material-related aroma in exhibiting relatively weak apparent roasting characteristics.

Overall, PLSR results indicated associations between sensory variation and aroma-active compound profiles across the commercial samples. Based on the PLSR distribution and OAV representativeness, 2-methylbutanal, pyrazines, and phenolic compounds showed potential associations with the observed roasting-related sensory differences. Pyrazines are closely associated with roasted and nutty aroma in sesame-related products, whereas phenolic compounds may contribute to burnt and smoky aroma characteristics under roasting conditions [15,38]. Some alcohols, aldehydes, and furans may be associated with grassy attributes in lightly roasted samples. However, PLSR mainly reflects correlations among variables and cannot directly prove actual sensory contributions of compounds. Therefore, the actual roles of related candidate compounds still need further verification by subsequent aroma addition experiments [49].

### 3.7. Sensory Validation of Candidate Aroma-Active Compounds by Aroma Addition

Based on GC-O, OAV, and PLSR results, an aroma-active compound addition experiment was conducted to evaluate the effects of candidate compounds on sensory attributes of sesame paste. Sample S5, assigned to the descriptive low apparent-roasting group, was used as the matrix. Four groups were prepared: control group (CK group), roasted nutty aroma-related compound group (R), burnt/smoky aroma-related phenolic compound group (B), and combined addition group (R+B). The results are shown in Figure 6B,C.

Compared with the CK group, roasted nutty scores of the R and R+B groups increased significantly from 3.62 to 6.92 (*p* < 0.05). The result indicated that the R mixture containing 2-methylbutanal and selected pyrazines enhanced roasted nutty aroma in sesame paste under the tested addition conditions. Pyrazines have been reported as important contributors to roasted and nutty aroma in sesame-related products [15,38].

The roasted nutty score of the B group was also higher than that of the CK group but lower than those of the R and R+B groups, indicating that phenolic compounds showed a weaker enhancement effect on roasted nutty aroma. For the burnt attribute, the B and R+B groups showed significantly higher scores than the CK group (*p* < 0.05), with the most pronounced increase observed in the B group. The B mixture enhanced the burnt attribute under the tested addition conditions, which was consistent with the reported burnt and smoky aroma characteristics of phenolic compounds in roasted sesame-related systems [15]. The R group showed a weaker enhancement effect on the burnt attribute, suggesting different sensory effects of the R and B mixtures. After addition treatments, typical sesame, grassy, and fatty scores decreased overall. The decrease in typical sesame score may be related to weakened perception of the original typical sesame aroma after enhanced roasted nutty and burnt aromas. Grassy score clearly decreased in all addition groups, with the largest decrease observed in the R+B group. Enhanced roasted nutty, burnt, and smoky aroma characteristics may mask grassy perception. The decrease in fatty score may also be associated with perceptual interactions among aroma compounds in the complex sesame paste matrix [53].

Overall, the addition experiment further supported the screening results from GC-O, OAV, and PLSR analyses. The R mixture containing 2-methylbutanal and pyrazines mainly enhanced roasted nutty aroma under the tested addition conditions, whereas the B mixture mainly enhanced burnt and smoky aroma characteristics. Combined addition enhanced roasted nutty and burnt attributes and reduced grassy perception. However, the addition experiment does not establish that the selected compounds are the sole contributors to the corresponding aroma attributes.

## 4. Conclusions

This study characterized color properties and aroma-related sensory and chemical profiles of commercial sesame paste samples exhibiting different apparent roasting characteristics. Darker samples generally showed higher browning indices, while stronger roasted nutty and burnt attributes and higher pyrazine levels were observed in products with more pronounced apparent roasting-related aroma characteristics. However, the color and aroma dimensions were not completely synchronous, as illustrated by S6. GC-O and OAV screening identified candidate aroma-active compounds, while exploratory PLSR indicated associations between these compounds and sensory attributes. In the aroma-addition experiment, the R mixture containing 2-methylbutanal and selected pyrazines enhanced roasted nutty aroma, whereas the B mixture containing guaiacol and 4-vinyl-2-methoxyphenol enhanced burnt/smoky aroma under the tested addition conditions. Overall, color/browning characteristics and aroma-active chemical profiles provide complementary information for characterizing apparent roasting-related differences and aroma quality among commercial sesame paste products. Because the samples were not produced under experimentally controlled roasting conditions, these associations should not be interpreted as direct measures or causal effects of actual roasting temperature or duration.

## Figures and Tables

**Table 1:** Color parameters and browning index of different commercial sesame pastes.

Commercial Sesame Paste Samples	$L^{*}$	$a^{*}$	$b^{*}$	∆E	BI
S1	65.02 ± 0.01 ^b^	2.06 ± 0.01 ^k^	15.69 ± 0.01 ^n^	2.30 ± 0.01 ^m^	65.64 ± 0.00 ^m^
S2	64.76 ± 0.02 ^c^	0.79 ± 0.00 ^n^	19.46 ± 0.01 ^k^	5.05 ± 0.01 ^k^	67.62 ± 0.01 ^j^
S3	64.24 ± 0.01 ^d^	1.37 ± 0.02 ^m^	17.1 ± 0.06 ^m^	3.25 ± 0.04 ^l^	66.40 ± 0.03 ^l^
S4	63.15 ± 0.01 ^e^	1.73 ± 0.02 ^l^	18.05 ± 0.06 ^l^	4.70 ± 0.05 ^j^	67.36 ± 0.04 ^k^
S5	65.98 ± 0.01 ^a^	0.29 ± 0.00 ^o^	14.59 ± 0.01 ^o^	0.01 ± 0.00 ^n^	64.20 ± 0.01 ^n^
S6	62.86 ± 0.00 ^f^	2.46 ± 0.06 ^j^	23.10 ± 0.06 ^j^	9.33 ± 0.04 ^i^	71.28 ± 0.02 ^i^
S7	58.07 ± 0.07 ^h^	5.12 ± 0.06 ^g^	33.20 ± 0.07 ^b^	20.80 ± 0.10 ^b^	83.57 ± 0.14 ^c^
S8	54.82 ± 0.10 ^l^	6.50 ± 0.03 ^c^	28.81 ± 0.03 ^g^	19.12 ± 0.09 ^a^	81.51 ± 0.10 ^g^
S9	56.57 ± 0.01 ^i^	5.04 ± 0.00 ^h^	30.83 ± 0.01 ^e^	19.36 ± 0.00 ^c^	81.94 ± 0.01 ^e^
S10	58.36 ± 0.00 ^g^	4.64 ± 0.00 ^i^	27.44 ± 0.01 ^i^	15.57 ± 0.01 ^d^	77.44 ± 0.01 ^h^
S11	52.48 ± 0.02 ^n^	7.31 ± 0.05 ^a^	29.89 ± 0.09 ^f^	21.58 ± 0.07 ^a^	84.67 ± 0.12 ^b^
S12	55.65 ± 0.02 ^k^	6.57 ± 0.01 ^b^	31.17 ± 0.03 ^d^	20.53 ± 0.04 ^c^	83.58 ± 0.05 ^c^
S13	58.10 ± 0.01 ^h^	5.83 ± 0.02 ^e^	34.91 ± 0.05 ^a^	22.49 ± 0.05 ^b^	85.74 ± 0.06 ^a^
S14	53.75 ± 0.03 ^m^	6.45 ± 0.02 ^d^	28.34 ± 0.03 ^h^	19.41 ± 0.04 ^c^	81.64 ± 0.05 ^f^
S15	56.26 ± 0.03 ^j^	5.52 ± 0.01 ^f^	31.48 ± 0.09 ^c^	20.18 ± 0.06 ^d^	83.05 ± 0.08 ^d^

Note: Statistical analysis was performed across all 15 commercial sesame paste samples. Different lowercase superscript letters within the same column indicate significant differences among samples (*p* < 0.05).

**Table 2:** Sensory evaluation scores of commercial sesame paste samples.

Sample	Roasted Nutty	Burnt	Typical Sesame	Grassy	Fatty
S1	2.69 ± 0.85 ^ef^	2.46 ± 1.05 ^f^	3.15 ± 1.14 ^ef^	7.23 ± 0.73 ^a^	2.85 ± 1.14 ^g^
S2	2.38 ± 0.65 ^fg^	2.46 ± 1.13 ^f^	2.62 ± 0.65 ^f^	6.69 ± 0.95 ^ab^	2.15 ± 1.21 ^h^
S3	2.46 ± 0.66 ^fg^	3.08 ± 1.19 ^def^	3.46 ± 0.97 ^e^	6.69 ± 1.03 ^ab^	3.00 ± 0.91 ^g^
S4	3.23 ± 0.73 ^e^	3.15 ± 0.80 ^de^	3.38 ± 0.96 ^e^	6.08 ± 1.04 ^c^	3.38 ± 0.65 ^g^
S5	2.08 ± 0.49 ^g^	2.62 ± 0.65 ^ef^	3.23 ± 0.93 ^ef^	6.77 ± 1.24 ^ab^	3.08 ± 1.12 ^g^
S6	3.08 ± 0.64 ^e^	3.46 ± 1.61 ^d^	4.15 ± 1.34 ^d^	6.31 ± 1.32 ^bc^	4.00 ± 1.00 ^f^
S7	6.15 ± 0.69 ^d^	6.31 ± 0.63 ^c^	6.15 ± 0.69 ^c^	2.69 ± 0.75 ^d^	6.08 ± 0.76 ^de^
S8	7.00 ± 0.82 ^ab^	6.46 ± 0.66 ^c^	6.92 ± 1.04 ^b^	1.92 ± 0.76 ^ef^	6.62 ± 0.65 ^bcd^
S9	6.38 ± 0.96 ^cd^	6.08 ± 0.86 ^c^	6.23 ± 1.09 ^c^	2.46 ± 0.66 ^de^	5.69 ± 0.63 ^e^
S10	6.85 ± 0.80 ^bc^	6.31 ± 0.63 ^c^	6.62 ± 1.04 ^bc^	2.15 ± 1.07 ^de^	6.23 ± 0.83 ^de^
S11	7.00 ± 1.00 ^ab^	8.00 ± 0.82 ^a^	6.77 ± 0.73 ^bc^	1.00 ± 0.41 ^g^	6.46 ± 0.52 ^cd^
S12	7.46 ± 1.27 ^a^	8.00 ± 0.71 ^a^	7.77 ± 0.73 ^a^	1.08 ± 0.49 ^g^	7.38 ± 0.77 ^a^
S13	7.00 ± 0.58 ^ab^	7.23 ± 0.73 ^b^	6.92 ± 0.76 ^b^	1.38 ± 0.51 ^fg^	6.92 ± 0.76 ^abc^
S14	7.00 ± 0.91 ^ab^	7.85 ± 0.80 ^ab^	6.77 ± 0.73 ^bc^	1.31 ± 0.63 ^g^	6.46 ± 0.88 ^cd^
S15	7.08 ± 1.19 ^ab^	7.77 ± 0.73 ^ab^	7.62 ± 0.51 ^a^	1.38 ± 0.65 ^fg^	7.08 ± 0.86 ^ab^

Note: Statistical analysis was performed across all 15 commercial sesame paste samples. Different lowercase superscript letters within the same column indicate significant differences among samples (*p* < 0.05).

**Table 3:** Calibration curves, R^2^ values, mean OAVs, and occurrence frequencies of OAV > 1 for aroma-active compounds in sesame paste samples in the descriptive apparent-roasting groups.

No.	Compound	Odor Threshold (mg/kg; Medium)	Standard Curve	R^2^	Mean OAV in L Group	Frequency of OAV > 1 in L Group (%)	Mean OAV in M Group	Frequency of OAV > 1 in M Group (%)	Mean OAV in H Group	Frequency of OAV > 1 in H Group (%)	Include Group(s)
1	2-methylbutanal	0.0022 (oil)	y = 0.1912x + 0.346	0.9999	2.79	40	58.06	100	142.48	100	L, M, H
2	α-pinene	0.2740 (oil)	y = 1.6857x + 0.0294	1	0.09	0	0.02	0	1.46	20	H
3	Hexanal	0.0750 (oil)	y = 0.4555x + 0.0165	0.9997	0.40	20	0.58	20	0.61	40	L, M, H
4	2-Pentylfuran	0.1000 (oil)	y = 1.2965x − 0.0314	1	1.08	40	0.60	40	0.95	20	L, M, H
5	2-methylpyrazine	0.0600 (water)	y = 1.2598x − 0.0438	1	0.00	0	2.30	60	4.00	100	M, H
6	p-cymene	0.0050 (water)	y = 2.6000x − 0.0308	1	0.45	20	0.00	0	1.64	20	L, H
7	(*E*)-2-octenal	0.0040 (oil)	y = 0.4198x + 0.0939	0.9981	0.20	0	0.57	20	0.00	0	M
8	1-Hexanol	0.4000 (oil)	y = 0.5978x + 0.0045	0.9998	0.28	0	0.14	0	0.15	0	Not included
9	2-ethyl-6-methylpyrazine	0.0400 (water)	y = 0.8041x − 0.2042	0.9999	0.21	0	3.09	60	5.46	80	M, H
10	2-ethyl-5-methylpyrazine	0.3200 (oil)	y =1.0759x − 0.0479	1	0.00	0	0.07	0	0.56	40	H
11	2,3,5-trimethylpyrazine	0.2900 (oil)	y = 0.9139x − 0.0094	0.9999	0.01	0	0.44	0	1.20	60	H
12	Nonanal	0.1500 (oil)	y = 1.0969x − 0.0494	1	0.06	0	0.09	0	0.19	0	Not included
13	2-ethyl-3,5-dimethylpyrazine	0.0018 (oil)	y = 0.9579x − 0.0142	0.9999	4.57	40	11.49	80	33.34	100	L, M, H
14	1-octen-3-ol	0.0010 (oil)	y = 1.7585x − 0.0249	1	18.71	40	0.00	0	0.00	0	L
15	3-ethyl-2,5-dimethylpyrazine	0.0240 (oil)	y = 0.6939x − 0.0175	1	0.00	0	8.32	100	21.41	100	M, H
16	1-heptanol	0.0054 (water)	y =1.2724x − 0.0469	1	0.58	40	0.98	20	0.00	0	L, M
17	Benzaldehyde	0.0600 (oil)	y = 0.9397x − 0.0112	1	0.42	20	0.58	20	0.83	40	L, M, H
18	1-octanol	0.0270 (oil)	y = 1.3511x − 0.0518	0.9999	0.49	0	0.31	0	0.22	0	Not included
19	2-Pentylpyridine	0.0050 (oil)	y = 2.5965x − 0.0296	0.9999	0.15	0	0.08	0	0.08	0	Not included
20	phenylacetaldehyde	0.0220 (oil)	y = 0.8078x + 0.0046	0.9998	0.01	0	0.63	20	0.70	20	M, H
21	guaiacol	0.0100 (oil)	y = 0.5489x − 0.0298	1	0.01	0	0.90	20	2.44	60	M, H
22	γ-undecalactone	0.0060 (oil)	y = 0.9543x − 0.0139	0.9999	0.08	0	0.25	0	0.57	20	H
23	4-ethyl-2-methoxyphenol	0.0500 (oil)	y = 1.0537x − 0.0711	0.9999	0.00	0	0.23	20	0.02	0	M
24	4-vinyl-2-methoxyphenol	0.0500 (oil)	y = 0.6638x − 0.0355	0.9999	0.05	0	1.20	40	2.80	100	M, H

Note: L, M, and H represent descriptive low, intermediate, and high apparent-roasting groups, respectively, and do not represent controlled roasting treatments. The mean OAV represents the arithmetic mean of OAVs from five samples within the same roasting group. Undetected compounds were included in the calculation as OAV = 0. The frequency of OAV > 1 represents the percentage of samples with OAV > 1 within the same roasting group. A compound was included as an OAV-screened candidate aroma-active compound in a given roasting group when OAV > 1 was observed in at least one sample within that group. Calibration curves were established using five-point calibration curves, where y represents the peak area ratio of the target compound to the internal standard, and x represents the concentration ratio of the target compound to the internal standard.

## Data Availability

The original contributions presented in this study are included in the article/Supplementary Material. Further inquiries can be directed to the corresponding authors.

## Supplementary Materials

The following supporting information can be downloaded at https://www.mdpi.com/article/10.3390/foods15183225/s1. Table S1. Sample codes and geographic origins of the 15 commercial sesame paste samples; Table S2. Sensory attributes, definitions, and reference samples used for sesame paste sensory evaluation; Table S3. Composition and supplemental levels of candidate aroma-active compounds used in the aroma-addition experiment; Table S4. Identification information and semi-quantitative concentrations of volatile compounds detected by HS-SPME-GC-MS in commercial sesame paste samples; Figure S1. Hierarchical clustering analysis of commercial sesame paste samples’ color/browning characteristics.
