# Supplementary material for: Color Darkening and Pyrazine Accumulation Associated with Apparent Roasting Intensity and Aroma Quality in Commercial Sesame Paste
Source: Foods. 2026 Sep 11;15(18):3225. doi: 10.3390/foods15183225 (PMC13606593; doi:10.3390/foods15183225)
Supplement: Supplementary file 1 [file foods-15-03225-s001.zip › foods-4510689-supplementary.pdf]

Table S1. Sample codes and geographic origins of the 15 commercial sesame paste samples

Sample ID	S1	S2	S3	S4	S5	S6	S7	S8	S9	S10	S11	S12	S13	S14	S15
Origin	USA	Greece	Jordan	Japan	Syria	Japan	Shandong, China	Henan, China	Shanxi, China	Henan, China	Hebei, China	Beijing, China	Shandong, China	Tianjin, China	Shaanxi, China

Table S2. Sensory attributes, definitions, and reference samples used for sesame paste sensory evaluation

Flavor attribute	Description	Reference sample
Roasted nutty	Aroma associated with roasted sesame seeds and nuts, characterized by warm, toasted and nutty	Medium-roasted sesame seeds; roasted peanuts and cashews
Burnt	Aroma associated with over-roasted or scorched materials, with acrid, charred	Over-roasted nuts (20 min at 200°C)
Typical sesame	Characteristic aroma of sesame paste or sesame seeds, showing typical sesame without obvious burnt or grassy characteristics	Commercial sesame paste with typical sesame aroma; lightly roasted sesame seeds
Grassy	Fresh plant-like aroma resembling cut grass or immature seeds	Ground raw sesame seeds (5 g)
Fatty	Fat-like aroma associated with lipid-rich matrices or heated sesame oil	Heated sesame oil (5 g)

Table S3. Composition and supplemental levels of candidate aroma-active compounds used in the aroma-addition experiment

Compound	Treatment	Supplemental concentration in sesame paste (μg/kg)	Amount added per 10.00 g sample (μg)	Concentration in aroma mixture (μg/mL)
2-methylbutanal	R, R+B	128.18	1.282	12.82
2-methylpyrazine	R, R+B	99.23	0.992	9.92
2-ethyl-6-methylpyrazine	R, R+B	88.95	0.890	8.90
2-ethyl-5-methylpyrazine	R, R+B	74.29	0.743	7.43
2,3,5-trimethylpyrazine	R, R+B	143.53	1.435	14.35
2-ethyl-3,5-dimethylpyrazine	R, R+B	24.84	0.248	2.48
3-ethyl-2,5-dimethylpyrazine	R, R+B	212.66	2.127	21.27
guaiacol	B, R+B	10.10	0.101	1.01
2-methoxy-4-vinylphenol	B, R+B	56.98	0.570	5.70

Note: The supplemental concentration of each compound was set at 10% of the difference between its mean concentration in the high apparent-roasting group and its original concentration in the selected base matrix. Base matrix: S5. Each 10.00 g sample received 100 μL of the corresponding aroma mixture.

Table S4. Identification information and semi-quantitative concentrations of volatile compounds detected by HS-SPME-GC-MS in commercial sesame paste samples

Compounds	CAS	Identification	RI a	RI b	Semi-quantitative concentrations of aroma compound(μg/kg)														
					S1	S2	S3	S4	S5	S6	S7	S8	S9	S10	S11	S12	S13	S14	S15
2-ethylhexanal	123-05-7	MS	-	670	2.25±0.1	3.89±2.39	3.7±3	1.76±0	-	0.57±0.14	-	1±0.84	1.06±0.77	1.23±1.23	3.9±3.24	8.8±6.87	-	2.74±1.51	-
heptane	142-82-5	MS	-	703	1.04±0.48	2.74±0.24	14.13±1.78	1.51±0.16	-	7.45±2.44	1.74±1.14	2.2±1.22	0.36±0.4	2.56±0.2	3.25±1.82	-	-	3.36±2.69	0.89±0.38
2-nonanone	821-55-6	MS/RI	770	739	-	-	-	-	-	-	-	2.31±0.87	-	-	8.35±3.83	-	-	-	-
octane	111-65-9	MS	-	795	18.68±24.54	3.34±2.59	5.94±0.71	1.3±0.67	0.89±0.1	6.69±1.37	1.79±1.12	-	0.26±0	3.02±2.11	15.68±0	-	-	5.04±0	-
2-methylbutanal	96-17-3	MS/RI/S	879	843	-	-	25.74±1.41	0.96±0.67	4.02±0.52	34.12±36.22	114.7±18.27	2.32±0.21	73.49±19.06	18.99±2.56	647.88±43.39	750.73±19.3	78.71±7.86	414.02±73.08	71.01±17.44
toluene	108-88-3	MS/RI	1019	1022	-	5.83±3.64	7.73±3.82	1.24±0.25	-	2.74±1.96	4.63±1.26	12.74±1.33	0.77±0.39	7.55±1.43	8.75±6.79	23.48±19.71	47.4±4.49	34.12±31.07	6.88±2.65
α-pinene	80-56-8	MS/RI/S	1008	1004	70.65±9.26	39.41±24.57	-	17.01±1.93	-	-	-	-	23.88±2.83	-	1835.29±1054.05	-	-	-	162.1±2.61
dimethyl disulfide	624-92-0	MS/RI	1037	1008	-	-	3.02±0.11	-	-	-	0.52±0	9.05±0.71	-	0.65±0.39	-	5.24±0.01	-	-	-
hexanal	66-25-1	MS/RI/S	1048	1033	-	-	-	5.38±0.77	146.41±6.24	-	28.41±1.02	-	-	-	122.38±52.07	85.89±4.07	-	188.09±5.79	18.43±1.36
p-xylene	95-47-6	MS/RI	1100	1091	-	12.85±10.5	-	13.17±1.22	12.04±3.39	-	-	-	-	-	-	-	36.39±2.97	4.39±3.65	-
pyridine	110-86-1	MS/RI	1150	1115	-	-	-	-	-	-	-	-	-	-	8.37±12.01	-	-	-	-
2-ethylpyrrole	1551-06-0	MS	-	1118	-	-	-	-	-	-	-	-	0.65±0.09	5.24±0.83	-	-	-	-	-
3-carene	13466-78-9	MS/RI	1130	1126	-	52.56±61.84	-	44.62±3.4	-	-	-	-	-	12.88±0.23	10.42±3.92	-	4.05±2.99	-	-
2-heptanone	110-43-0	MS/RI	1173	1135	-	1.16±0.57	33.64±2.75	-	14.12±0.24	44.72±0	8.75±0.67	-	12.42±3.36	6.85±0.82	-	-	55.11±2.16	-	37.3±4.09

2-methyl-1-butanol	137-32-6	MS/RI	1191	1154	86.15±6.64	114.24±31.57	-	3.62±1.22	8.96±0.2	199.69±6.56	-	-	-	2.36±0.51	42.4±21.4	-	-	-	-
2-methylthiazole	3581-87-1	MS	-	1169	-	-	-	-	-	-	2.17±0.38	-	-	-	-	15.8±0.8	-	-	-
limonene	138-86-3	MS/RI	1176	1171	59.59±2.76	2.35±0.86	0.7±0.45	1.08±0.24	-	-	-	-	2.15±0.06	1.79±0.93	24.79±19.72	-	14.99±5.97	-	42±0.79
2-pentylfuran	3777-69-3	MS/RI/S	1200	1182	160.4±7.76	87.59±20.63	168.12±7.08	37.83±3.85	85.1±9.99	144.73±8.65	24.05±1.51	-	24.54±1.26	36.22±4.15	231.4±26.56	54.38±6.76	94.56±8.92	105.16±15.54	60.51±2.46
2-methylpyrazine	109-08-0	MS/RI/S	1236	1192	-	-	-	-	-	142±12.53	40.83±0.81	239.7±40.4	49.82±4.95	60.92±7.39	425.61±20.07	324.02±18.68	268.3±24.13	218.75±17.98	119.98±2
ocimene	13877-91-3	MS/RI	1233	1203	142.18±16.39	742.45±446.45	376.54±26.96	93.84±12.89	282.83±12.35	266.23±28.51	127.82±2.14	60.9±33.52	254±84.95	116.34±10.2	238.85±25.65	120.43±9.71	69.38±8.32	109.73±10.82	24.51±2.63
dodecane	112-40-3	MS	-	1227	-	-	-	-	286.34±9.48	-	-	109.76±57.29	-	-	-	-	1.05±0.02	407.53±58.6	-
p-cymene	99-87-6	MS/RI/S	1244	1230	8.75±0	-	-	2.39±0.85	-	-	-	-	-	-	37.06±13.21	-	-	-	4.13±0.32
2-octanone	111-13-7	MS/RI	1276	1235	-	-	8.94±3.44	-	-	-	1.01±0	-	-	-	-	1.99±0.83	1.88±1.24	-	-
octanal	124-13-0	MS/RI	1264	1242	-	-	5.18±3.34	-	10.39±3.97	4.7±0.82	2.9±0.21	-	2.23±0.43	-	7.8±0.33	3.92±0.59	1.46±1.09	17.03±19.24	3.45±0.48
(E)-2-octenal	2548-87-0	MS/RI/S	1270	1248	3.01±0.28	-	-	1.02±0.46	-	11.47±1.24	-	-	-	-	-	-	-	-	-
2,5-dimethylpyrazine	123-32-0	MS/RI	1300	1255	24.5±1.66	19.75±22.74	10.63±0.76	27.54±0.81	7.74±0.35	298.48±78.09	148.69±3.69	257.29±47.23	113.12±12.84	187.63±29.39	1035.39±3.24	920.03±45.73	838.47±58.78	534.64±37.3	217.08±5.02
2,6-dimethylpyrazine	108-50-9	MS/RI	1305	1261	-	-	-	-	-	30.85±2.35	17.79±1.49	44.84±4.8	33.33±1.92	20.48±2.27	165.65±4.56	153.58±7.71	110.69±9.15	65.4±38.15	29.81±2.35
ethylpyrazine	13925-00-3	MS/RI	1310	1261	-	-	-	2.33±1.2	-	28.68±2.38	14.62±0.59	33.37±8.45	15.2±1.37	15.6±4.12	124.9±7.89	101.96±5.89	66.19±4.36	68.45±3.34	29.21±2.36
2,3-dimethylpyrazine	5910-89-4	MS/RI	1316	1277	-	-	-	-	-	54.13±11.9	18.59±0.87	-	-	29.72±1.7	142.71±2.65	115.25±3.88	96.11±2.06	70.77±20.91	26.98±2.41
1-hexanol	111-27-3	MS/RI/S	1320	1287	371.3±14.82	108.91±45.17	0.44±0.08	56.51±3.01	32.21±0.84	230.4±18.1	1.94±0.15	-	4.43±0.44	7.89±0.38	155.01±9.21	-	28.69±1.91	49.63±23.3	15.45±3.97

2-ethyl-2-hexenal	645-62-5	MS/RI	1330	1303	0.45±0.04	-	-	-	2.89±0.4	2.93±1.96	1.63±0.1	-	1.09±0.62	1.61±0.69	8.19±1.6	7.2±1.61	3.58±0.61	-	2.16±1.09
2-ethyl-6-methylpyrazine	13925-03-6	MS/RI/S	1345	1312	8.91±1.62	18.59±0	-	11.11±1.87	3.56±1.54	104.06±47.19	27.16±3.45	132.05±48.28	16.46±4.38	37.11±4.53	624.51±22.64	228.82±19.48	133.88±8.19	337.43±171.54	68.09±52.43
2-ethyl-5-methylpyrazine	13360-64-0	MS/RI/S	1356	1328	-	-	5.47±1.86	-	-	-	61.59±8.54	-	49.01±6.77	79.87±9.74	-	371.87±27.17	354.45±48.13	-	91.27±4.96
neoalloocimene	7216-56-0	MS	-	1334	10.12±8.17	75.32±30.69	29.95±12.56	8.28±2.1	12.53±4	10.32±7.63	16.18±8.31	-	83.66±114.09	10.25±8.92	-	-	-	-	-
2,3,5-trimethylpyrazine	14667-55-1	MS/RI/S	1385	1339	5.47±3.51	-	-	9.52±3.36	1.95±1.32	96.67±51.77	84.9±11.69	142.7±43.35	48.67±4.57	128.86±17.06	496.86±68.64	482.2±24.29	507.65±65.8	267.4±114.59	128.23±7.07
nonanal	124-19-6	MS/RI/S	1367	1347	26.99±7.66	9.1±6.25	12.54±6.05	-	-	30.88±5.94	14.23±7.23	-	6.85±1.63	8.81±1.88	-	14.82±6.77	18.39±7.59	13.81±10.51	42.91±3.12
acetic acid	64-19-7	MS/RI	1400	1354	-	-	-	-	25.36±4.78	27.45±2.74	-	-	-	-	26.28±1.44	14.67±7.74	-	16.29±7.14	8.17±2.46
DL-2-octanol	123-96-6	MS/RI	1411	1361	1.88±1.08	11.17±9.6	-	1.05±1.17	-	-	-	-	-	-	-	-	-	-	-
furfural	98-01-1	MS/RI	1415	1369	-	-	-	0.62±0	0.78±0.18	5.08±1.28	-	-	1.8±0.48	-	12.53±1.57	-	-	-	4.12±0.63
2-ethyl-3,5-dimethylpyrazine	13925-07-0	MS/RI/S	1420	1373	-	15.74±0	-	25.36±3.07	-	38.55±20.3	20.77±0.99	-	14.73±2.08	29.98±7.3	124.86±32.46	122.54±22.77	18.19±2.21	29.36±34.49	4.44±0.49
1-octen-3-ol	3391-86-4	MS/RI/S	1399	1384	-	-	66±3.92	-	27.53±1.71	-	-	-	-	-	-	-	-	-	-
3-ethyl-2,5-dimethylpyrazine	13360-65-1	MS/RI/S	1416	1385	-	-	-	-	-	218.27±2.49	140.21±8.55	179.99±34.66	76.53±9.23	181.48±30.01	754.66±149.41	559.14±95.5	841.47±73.41	383.11±105.64	232.42±11.38
1-heptanol	111-70-6	MS/RI/S	1427	1388	-	7.02±5.6	-	-	8.53±1.88	26.51±0	-	-	-	-	-	-	-	-	-
DL-isoborneol	124-76-5	MS/RI	1452	1413	-	-	10.03±0.39	-	-	-	8.07±0.55	8.89±0.62	8.54±1.25	8.34±3.29	36.71±8.48	30.13±2.09	47.18±3.25	15.74±8.21	8.92±0.35
2-ethylhex-2-enol	50639-00-4	MS	-	1416	-	7.16±5.43	-	-	2.05±0.81	-	-	-	-	-	-	-	-	-	-
2-(prop-1-en-2-yl)pyrazine	38713-41-6	MS	-	1418	-	-	-	-	-	-	3.89±0.04	-	4.22±0	5.2±1.28	26.34±9.77	28.08±1.85	-	-	9.8±0.56

benzaldehyde	100-52-7	MS/RI/S	1464	1425	-	1.45±0.66	15.29±1.69	0.93±0.52	107.56±7.04	88.63±11.02	16.91±1.08	12.57±0.82	31.22±4.14	9.4±1.32	95.78±3.83	89.3±9.3	27.88±3.18	23.64±7.35	25.44±0.53
2-ethylhexanol	104-76-7	MS/RI	1470	1425	4.75±0.41	11.06±2.29	-	7.14±0.69	-	-	-	24.2±1.96	-	-	-	79.25±5.47	166.91±24.31	90.8±8	-
3,5-diethyl-2-methylpyrazine	18138-05-1	MS/RI	1469	1438	-	-	-	-	-	-	11.79±2.32	9.28±4.06	-	17.83±4.62	74.75±23.67	58.46±16.63	67.38±13.72	32.09±2.14	18.52±0.76
(E,E)-3,5-octadien-2-one	30086-02-3	MS/RI	1470	1447	1.89±0.36	-	-	-	15.16±2.53	3.71±0.67	-	2.34±0.69	-	1.47±0.1	11.98±0.67	-	5.61±1.42	-	-
3,5-dimethyl-2-propylpyrazine	32350-16-6	MS	-	1461	-	-	-	-	-	-	-	-	-	6.71±1.49	-	-	36.23±0	-	10.38±0.09
2-methyl-5-[(E)-1-propenyl]pyrazine	55138-64-2	MS/RI	1500	1469	-	-	-	-	-	24.18±7.41	12.67±1.7	12.39±1.65	6.68±0.68	13.3±1.63	51.16±8.26	44.43±2.12	51.45±1.51	31.48±7.7	14.9±2.81
3-methylpyrrole	616-43-3	MS/RI	1520	1475	-	-	-	-	-	-	2.85±0.96	4.17±0.21	2.19±1.1	2.66±0.07	22.91±3.1	17.38±1.08	3.86±0.17	-	9.68±2.05
1-octanol	124-11-8	MS/RI/S	1525	1490	22.68±2.56	8.3±8.58	8.05±1.17	7.97±0.61	18.49±4.13	23.94±4.61	2.34±0.18	2.28±0.58	3.57±0.65	2.29±0.83	14.23±4.18	-	12.95±4.42	10.23±2.98	-
tetrahydro-2-(2-propynyloxy)-2H-pyran	6089-04-9	MS	-	1509	2.5±0.41	-	-	-	-	9±0.79	-	-	1.3±0.31	0.91±0.13	-	-	12.64±1.44	9.15±0.56	3.14±0.67
2-pentylpyridine	2294-76-0	MS/RI/S	1527	1514	1.8±1.16	-	1.91±0.05	-	-	-	2.02±0.67	-	-	1.94±0	-	-	-	-	-
γ-butyrolactone	96-48-0	MS/RI	1570	1530	1.14±0.55	4.52±0	0.86±0.32	1.45±0.16	-	18.51±3.5	-	6.41±1.41	4.23±0.95	6.46±0.84	38.93±10.46	26.15±4.32	-	29.86±4.49	13.66±0.53
3-nonen-2-one	18402-83-0	MS/RI	1523	1534	-	-	-	-	-	-	0.53±0.44	8.69±0.74	0.49±0.28	1±0.31	11.18±5.94	6.1±2.45	2.31±0.47	-	-
(E)-2-octen-1-ol	18409-17-1	MS/RI	1618	1587	12.25±1.54	-	0.81±0.02	1.06±0.38	1.24±0.04	-	-	-	-	1.45±0.22	-	-	2.29±1.01	-	0.84±0.32
phenylacetaldehyde	122-78-1	MS/RI/S	1617	1596	-	-	-	1.36±0.53	-	16.22±0.67	9.64±1.09	6.09±1.96	4.89±1.32	3.03±0.91	15.94±1.14	37.08±4.77	10.31±0.55	31.87±9.42	10.95±3.22
2-acetylthiazole	24295-03-2	MS/RI	1641	1601	-	-	-	-	-	-	-	0.93±1.19	-	-	3.52±3.05	-	-	-	-
2-furanmethanol	98-00-0	MS/RI	1620	1604	-	-	-	0.25±0	-	3.25±0.88	0.37±0.09	5.24±2.26	2.96±1.71	1.2±0.45	21.51±3.93	7.63±4.01	4.11±0.67	12.13±6.17	5.5±2.15

mesitylene	108-67-8	MS/RI	1587	1559	-	-	-	0.87±0.18	-	-	2.9±0.06	-	-	3.38±1.81	20.09±3.76	-	-	12.62±4.58	5.22±1.71
acetophenone	98-86-2	MS/RI	1607	1609	0.89±0.23	1.24±0.94	1.12±0.06	-	0.84±0.05	5.07±0.62	-	15.04±1.92	2.05±1.22	-	-	19.6±3.02	8.27±1.01	-	-
farnesene	502-61-4	MS/RI	1615	1620	-	25.97±0.73	8.78±1.57	-	8.84±0.46	8.31±0.54	-	2.87±1.21	5.48±1.05	6.04±1.98	15.61±10.31	16.21±7.31	16.52±3.19	24.89±9	11.28±1.73
2-methylbutanoic acid	116-53-0	MS/RI	1641	1628	2.32±1.57	-	-	-	-	10.95±3.33	-	-	4.74±2.57	-	-	-	-	-	-
1-nonanol	143-08-8	MS/RI	1619	1630	20.28±3.06	-	-	8.33±1.69	4.28±1.06	11.39±3.46	-	-	-	-	-	-	6.78±0.75	-	2.23±0.36
γ-hexalactone	695-06-7	MS/RI	1664	1662	4.55±0.73	1.83±1.93	1.12±0.15	0.48±0.16	1.93±0.21	11.1±3.5	-	4.88±0	2.67±0.73	2.38±1.6	25.51±5.6	-	14.92±4.15	15.07±2.43	7.97±1.09
5-methyl-2-furanmethanol	3857-25-8	MS/RI	1720	1698	-	-	-	-	-	-	-	6.82±0.57	-	-	-	-	-	-	1.94±0.87
1,2-dimethoxybenzene	91-16-7	MS/RI	1706	1710	1.45±0.76	-	0.72±0.46	0.59±0	-	1.8±0.52	0.3±0	-	-	0.75±0.84	-	-	2.34±0.16	19.55±3.64	1.28±0
4-allylphenol	501-92-8	MS	–	1715	-	-	-	-	-	-	2.67±1.67	-	0.52±0	2.98±2.21	13.94±7.1	10.65±10.34	10.23±3.7	9.28±0.41	5.37±0
naphthalene	91-20-3	MS/RI	1733	1740	1.68±0.27	3.67±1.27	0.82±0.09	0.95±0.22	1.79±0.53	1.78±1.01	0.56±0	1.15±0.16	0.52±0.05	0.71±0.11	-	-	-	-	-
methyl nicotinate	93-60-7	MS	–	1745	-	-	-	-	-	-	-	-	-	0.87±0.2	4.2±3.24	-	-	-	0.99±0.25
1-pentyl-1H-pyrrole	699-22-9	MS	–	1750	-	-	-	-	-	-	-	-	-	0.67±0.39	9.21±2.13	2.81±0.6	-	3.99±0.75	-
1-furfurylpyrrole	1438-94-4	MS	–	1770	-	-	-	-	-	-	-	-	-	-	-	1.15±0.58	-	-	-
(*E*,*E*)-2,4-decadienal	25152-84-5	MS/RI	1789	1780	-	-	-	-	-	-	-	2.03±0.91	-	-	12.4±3.72	-	2.02±0.29	2.24±1.15	-
hexanoic acid	142-62-1	MS/RI	1810	1814	16.55±2.71	-	2.56±0.19	-	28.56±11.46	8.75±0.72	9.57±0.28	-	-	12.57±1.12	59.78±13.84	-	-	59.02±21.63	50.43±0.21
guaiacol	90-05-1	MS/RI/S	1823	1816	-	-	-	0.38±0	-	8.29±6.63	0.64±0.09	24.08±2.12	9.08±1.74	0.86±0.56	2.58±3.49	48.76±1.66	49.98±15.57	2.92±0.67	19.87±0

benzyl alcohol	100-51-6	MS/RI	1837	1840	4.64±0.7	3.95±1.85	-	1.94±0.04	-	6.8±0.66	1.87±0.3	3.66±0.57	2.08±0.01	2.45±0.49	11.53±3.32	3.59±1.1	4.05±0.68	11.13±3.2	4.68±0.27
phenethyl alcohol	60-12-8	MS/RI	1873	1880	23.8±4.37	8.05±8.84	-	4.53±1.3	-	22.83±5.78	2.45±0.4	4.54±0.52	3.43±0.59	4.5±1.3	21.44±1.93	-	6.89±0.79	42.4±11.12	6.94±0.42
γ-undecalactone	104-67-6	MS/RI/S	–	1885	1.39±0.19	-	1.07±0.48	0.45±0.32	0.84±0.55	5.16±5	-	-	0.82±0.58	1.89±0.69	11.01±6.32	2.14±0	4.97±3.07	6.45±1	8.73±5.96
α-ethylidenebenzeneacetaldehyde	4411-89-6	MS/RI	1907	1926	-	-	-	-	-	9.41±5	11.77±4.5	5.69±1.12	8.43±5.16	2.37±1.04	40.99±7.18	35.88±8.59	14.59±1.7	42.98±9.89	10.82±3.79
2-ethylhexanoic acid	149-57-5	MS/RI	1969	1953	-	-	-	0.08±0	-	-	4.24±0	0.78±0	-	-	-	3.36±3.04	-	50.71±10.04	-
phenol	108-95-2	MS/RI	1950	1977	0.99±0.14	-	-	-	-	-	0.19±0.03	0.7±0.22	0.35±0.13	0.23±0.11	1.45±0.25	1.31±0.48	-	0.91±0.17	0.96±0.11
pyrrole-2-carboxaldehyde	1003-29-8	MS/RI	2006	1992	-	-	-	-	-	-	-	0.81±0.21	-	-	2.27±0.54	1.03±0.18	-	-	-
DL-pantolactone	79-50-5	MS	–	1998	-	-	-	2.63±0.37	-	14.68±0.85	5.96±3.73	2.16±0.94	4.6±2.7	5.14±3.68	30.37±15.96	6.9±6.66	12.04±4.77	32.77±6.52	8.37±3.74
furaneol	3658-77-3	MS/RI	2002	2004	-	-	-	-	-	-	1.32±0.92	-	1.04±0.84	-	-	2.5±0.01	-	7.33±4.1	-
4-ethyl-2-methoxyphenol	2785-89-9	MS/RI/S	2033	2014	-	-	-	-	-	-	56.33±10.16	0.29±0.13	-	0.62±0.26	2.93±0.13	-	0.61±0.12	-	-
N-methylpyrrole-2-carboxaldehyde	1192-58-1	MS	–	2050	-	-	-	-	-	-	-	0.22±0.02	-	-	1.01±0.31	0.64±0.16	-	-	-
2-phenoxyethanol	122-99-6	MS/RI	2107	2111	-	-	-	-	-	-	-	0.59±0.32	-	-	-	1.26±1.04	-	-	-
4-vinyl-2-methoxyphenol	7786-61-0	MS/RI/S	2151	2162	1.18±0.06	1.58±1.34	4.85±2.5	2.50±0.38	2.32±0.26	22.77±8.79	-	46.64±10.7	61.14±15.52	55.72±24.24	227.25±53.28	135.5±52.19	180.34±26.75	168.93±48.05	101.13±11.75

Note: MS, identification based on mass spectral matching with the NIST library; RIa, retention index obtained from the NIST library database; RIb, retention index calculated using a homologous series of n-alkanes (C7–C40); S, identification confirmed using authentic standards. Compounds without authentic standard confirmation were tentatively identified based on the combined evidence of mass spectra and retention indices. Volatile compound concentrations were semi-quantitatively estimated using 1,2-dichlorobenzene as an internal reference and are expressed as mean ± standard deviation from triplicate measurements. These semi-quantitative concentrations were used for comparative volatile profiling among samples and were not directly used for OAV calculation. "–" indicates not detected.

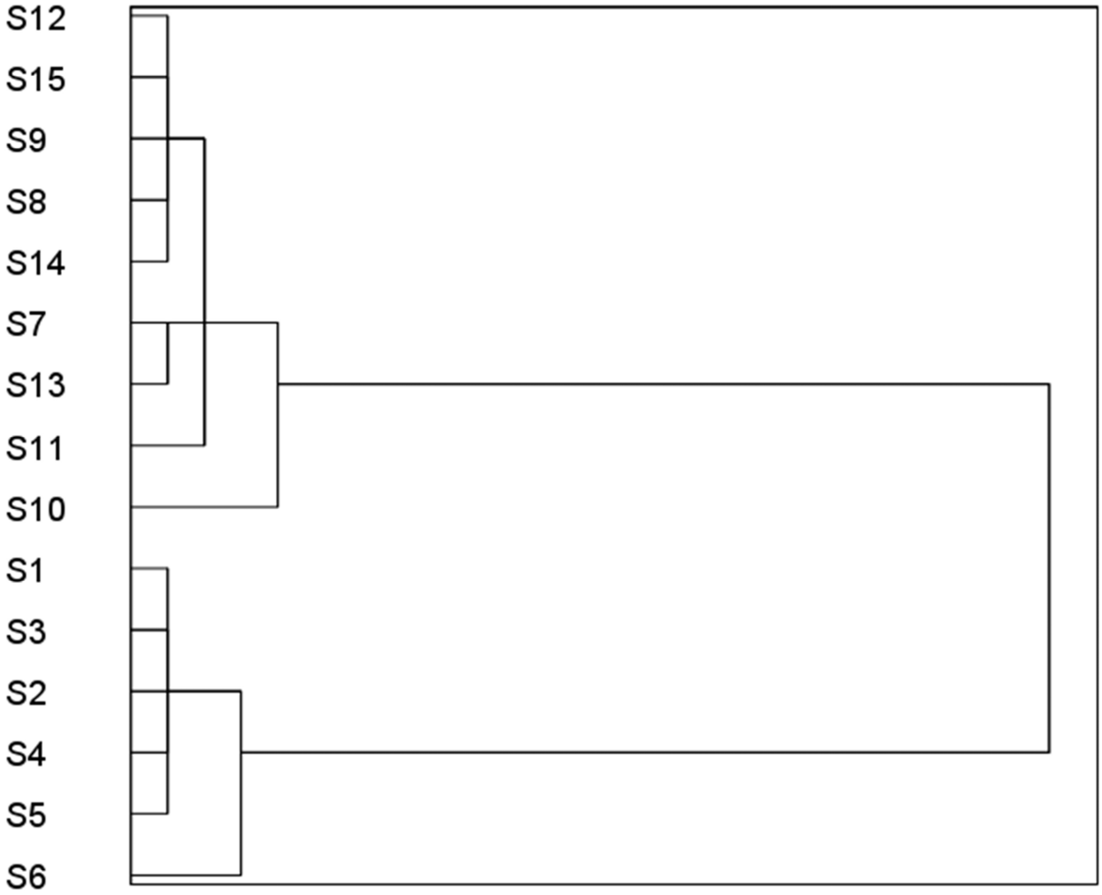

Figure S1. Hierarchical clustering analysis of commercial sesame paste samples color/browning characteristics.
